# Multi-scale evidence for declining microbial carbon fixation along forest succession gradients

**DOI:** 10.1093/ismejo/wraf191

**Published:** 2025-08-24

**Authors:** Shu-Yi-Dan Zhou, Zhiyang Lie, Chaotang Lei, Qi Zhang, Xujun Liu, Guopeng Wu, Roy Neilson, Fu-Yi Huang, Guowei Chu, Ze Meng, Dong Zhu, David T Tissue, Josep Peñuelas, Juxiu Liu

**Affiliations:** Guangdong Provincial Key Laboratory of Applied Botany, South China Botanical Garden, Chinese Academy of Sciences, 723 Xingke Road, Tianhe District, Guangzhou 510650, China; National Ecological Science Data Center Guangdong Branch, South China Botanical Garden, Chinese Academy of Sciences, 723 Xingke Road, Tianhe District, Guangzhou 510650, China; Guangdong Province Data Center of Terrestrial and Marine Ecosystems Carbon Cycle, South China Botanical Garden, Chinese Academy of Sciences, 723 Xingke Road, Tianhe District, Guangzhou 510650, China; Guangdong Provincial Key Laboratory of Applied Botany, South China Botanical Garden, Chinese Academy of Sciences, 723 Xingke Road, Tianhe District, Guangzhou 510650, China; National Ecological Science Data Center Guangdong Branch, South China Botanical Garden, Chinese Academy of Sciences, 723 Xingke Road, Tianhe District, Guangzhou 510650, China; Guangdong Province Data Center of Terrestrial and Marine Ecosystems Carbon Cycle, South China Botanical Garden, Chinese Academy of Sciences, 723 Xingke Road, Tianhe District, Guangzhou 510650, China; Guangdong Provincial Key Laboratory of Applied Botany, South China Botanical Garden, Chinese Academy of Sciences, 723 Xingke Road, Tianhe District, Guangzhou 510650, China; National Ecological Science Data Center Guangdong Branch, South China Botanical Garden, Chinese Academy of Sciences, 723 Xingke Road, Tianhe District, Guangzhou 510650, China; Guangdong Province Data Center of Terrestrial and Marine Ecosystems Carbon Cycle, South China Botanical Garden, Chinese Academy of Sciences, 723 Xingke Road, Tianhe District, Guangzhou 510650, China; Institute for Advanced Study, Shaoxing University, Shaoxing 312000, China; Guangdong Provincial Key Laboratory of Applied Botany, South China Botanical Garden, Chinese Academy of Sciences, 723 Xingke Road, Tianhe District, Guangzhou 510650, China; National Ecological Science Data Center Guangdong Branch, South China Botanical Garden, Chinese Academy of Sciences, 723 Xingke Road, Tianhe District, Guangzhou 510650, China; Guangdong Province Data Center of Terrestrial and Marine Ecosystems Carbon Cycle, South China Botanical Garden, Chinese Academy of Sciences, 723 Xingke Road, Tianhe District, Guangzhou 510650, China; Guangdong Provincial Key Laboratory of Applied Botany, South China Botanical Garden, Chinese Academy of Sciences, 723 Xingke Road, Tianhe District, Guangzhou 510650, China; National Ecological Science Data Center Guangdong Branch, South China Botanical Garden, Chinese Academy of Sciences, 723 Xingke Road, Tianhe District, Guangzhou 510650, China; Guangdong Province Data Center of Terrestrial and Marine Ecosystems Carbon Cycle, South China Botanical Garden, Chinese Academy of Sciences, 723 Xingke Road, Tianhe District, Guangzhou 510650, China; Ecological Sciences, The James Hutton Institute, Dundee DD2 5DA, United Kingdom; State Key Laboratory of Regional and Urban Ecology, Ningbo Observation and Research Station, Institute of Urban Environment, Chinese Academy of Sciences, Xiamen 361021, China; Guangdong Provincial Key Laboratory of Applied Botany, South China Botanical Garden, Chinese Academy of Sciences, 723 Xingke Road, Tianhe District, Guangzhou 510650, China; National Ecological Science Data Center Guangdong Branch, South China Botanical Garden, Chinese Academy of Sciences, 723 Xingke Road, Tianhe District, Guangzhou 510650, China; Guangdong Province Data Center of Terrestrial and Marine Ecosystems Carbon Cycle, South China Botanical Garden, Chinese Academy of Sciences, 723 Xingke Road, Tianhe District, Guangzhou 510650, China; Guangdong Provincial Key Laboratory of Applied Botany, South China Botanical Garden, Chinese Academy of Sciences, 723 Xingke Road, Tianhe District, Guangzhou 510650, China; National Ecological Science Data Center Guangdong Branch, South China Botanical Garden, Chinese Academy of Sciences, 723 Xingke Road, Tianhe District, Guangzhou 510650, China; Guangdong Province Data Center of Terrestrial and Marine Ecosystems Carbon Cycle, South China Botanical Garden, Chinese Academy of Sciences, 723 Xingke Road, Tianhe District, Guangzhou 510650, China; State Key Laboratory of Regional and Urban Ecology, Ningbo Observation and Research Station, Institute of Urban Environment, Chinese Academy of Sciences, Xiamen 361021, China; Hawkesbury Institute for the Environment, Western Sydney University, Hawkesbury Campus, Richmond NSW 2753, Australia; CSIC, Global Ecology Unit CREAF- CSIC-UAB, Bellaterra, Barcelona 08193, Catalonia, Spain; CREAF, Cerdanyola del Vallès, Barcelona 08193, Catalonia, Spain; Guangdong Provincial Key Laboratory of Applied Botany, South China Botanical Garden, Chinese Academy of Sciences, 723 Xingke Road, Tianhe District, Guangzhou 510650, China; National Ecological Science Data Center Guangdong Branch, South China Botanical Garden, Chinese Academy of Sciences, 723 Xingke Road, Tianhe District, Guangzhou 510650, China; Guangdong Province Data Center of Terrestrial and Marine Ecosystems Carbon Cycle, South China Botanical Garden, Chinese Academy of Sciences, 723 Xingke Road, Tianhe District, Guangzhou 510650, China

**Keywords:** old-growth forests, isotope labelling, carbon cycle, microbial network, plant richness

## Abstract

Although soil carbon accumulates during subtropical forest succession, changes in microbial communities and their carbon fixation capacity remain unclear. Using an integrative approach that combines field experimentation, extensive global metagenomic data, and isotope labelling, we analysed 84 soil microbiomes from a long-term successional site and 755 global metagenomes to investigate microbial community dynamics and their role in carbon fixation. Based on field data, bacteria, fungi, and protists had synchronous succession with vegetation; however, the relative abundance of carbon fixation genes declined significantly in later successional stages. To further investigate this outcome, we analysed global data from planted and mature natural forests and found significantly higher carbon fixation potential in planted forests, predominantly driven by *Pseudomonadota* and *Actinomycota* members. Field-based ^13^C labelling results further confirmed a significant decline in microbial CO₂ fixation rates with forest succession. These findings underscore the ecological importance of microbial carbon fixation in early forest succession, emphasizing its foundational role in initiating soil carbon accumulation and shaping long-term carbon cycling trajectories.

## Introduction

Forest ecosystems cover >30% of the total global land area and represent a key carbon (C) reservoir [1, 2]. The effective restoration of forests depends on natural successional processes to increase species diversity that leads to the gradual recovery of ecosystem functions [3, 4] and stability [5]. For example, successional processes drive transitions in forest vegetation communities, where tree diversity of tropical and subtropical forests increases with succession, leading to greater litter biomass and below-ground root exudation [6, 7]. Shifts in forest vegetation, along with associated soil nutrient content [8], lead to concomitant successional driven changes in microbial biomass and community structure, through species recruitment and diversification in interaction networks [9, 10], particularly altering the soil microbiome [11]. As a key driver of ecosystem stability the soil microbiome has the capacity to buffer environmental disturbances, including those caused by global change [12]. Vegetation and soil microbial communities are interlinked and exhibit feedback effects, suggesting that microbial communities during long-term forest succession may mirror stages of plant succession [13], or in other words, above- (plants) and below-ground (microorganisms) components of forest ecosystems undergo synchronous succession. However, there is limited research on this topic, especially regarding the dynamics of microbial communities and interaction networks during forest succession, which are crucial for gaining deeper insights into the functioning of soil microbial communities.

Forest ecosystem services interact with and benefit other terrestrial ecosystems [14], largely due to the diversity of bacteria, fungi, and protozoa [15] and their roles in biogeochemical cycles [16]. For example, autotrophic forest soil microbial communities are involved in CO_2_ fixation, with implications for carbon sequestration [17]. Moreover, previous studies have suggested significant contributions of microbial carbon fixation in aquatic ecosystems [18, 19]. However, there are microorganisms in the soil that rely solely on CO_2_ as their carbon source, and these microorganisms are understudied [20]. Currently, microbial CO_2_ fixation efficiency is mainly assessed through analysing the abundance of specific marker genes or isotopically labelled compounds in cultured microorganisms [21]. Using such methodology has estimated that soil microorganisms may contribute ~4% of the total CO_2_ fixation in terrestrial ecosystems annually [22]. Thus, microbial CO_2_ fixation processes may influence the quantification of soil carbon balance. Microbial carbon fixation can be achieved through various metabolic pathways, such as the Arnon–Buchanan cycle [23], the Wood–Ljungdahl pathway [24], and the Calvin cycle [25]. When there is insufficient organic carbon, complex carbon sources, or imbalanced carbon demands due to rapid microbial growth, the importance of metabolic pathways increase, and microorganisms may re-fix CO_2_ produced by respiration to mitigate carbon loss [26]. However, due to the limited knowledge at global scale on this topic, the universality of this process remains uncertain. Although functional annotation of forest soil metagenomes provides useful context, it is an activity-based measure such as stable isotope labelling that offers the most direct and robust evidence of microbial function. Studying carbon fixation during forest succession helps reveal the potential discrepancies between inferred functional potential and real-time metabolic processes. Furthermore, the general focus of research on carbon metabolism processes mediated by microorganisms [27, 28] has created a gap in assessing microbial carbon fixation capacity under forest succession.

Whereas microbial functions in forest soils are known to be influenced by multiple biotic and abiotic drivers, such as nitrogen enrichment [29] and plant disease pressure [30] under changing climatic conditions, the role of long-term forest succession in shaping soil microbial networks and functions remains unclear. Therefore, we quantified subtropical forest soil microbial community structure and function at contrasting stages of succession in southern China. In addition, we collated 755 forest soil metagenomes from natural and planted forests, to annotate microbial carbon fixation pathways. By comparing the global data with experimental site data, we aimed to test the following hypotheses that (i) subtropical forest soil microbial community succession would reflect stages of above-ground plant succession, (ii) complexity and resilience of forest soil microbial networks would increase during succession, and (iii) increased inputs of soil organic carbon (SOC) during forest succession may affect microbial carbon fixation functional potential.

## Materials and methods

### Study sites and sampling

The forest successional field study sites were located within the Dinghu Mountain National Nature Reserve (23°09′ 21″ N, 112°30′ 39″ E) in Guangdong Province, China. It encompasses four successional stages of ecosystem development from primary to advanced communities: Pine Forest (S1, about ~60 years old), Mixed Pine and Broadleaf Forest (S2, about ~100 years), Mature Mixed Pine and Broadleaf Forest (S3, about ~150 years), and Monsoon Evergreen Broadleaf Forest (S4, > 400 years). Site information is provided in Note S1. The understory vegetation for each forest successional stage has been reported previously [31]. Each plot was subdivided into 25 sub-plots (20 × 20 m). In each forest successional stage, six sub-plots were randomly selected for assessment of understory vegetation richness and collection of soil and litter samples. A five-point composite sampling method was employed in each sub-plot. Topsoil (0–10 cm) was collected using an earth auger, sieved (2 mm) to remove stones, roots, and soil fauna, and stored at −20°C.

### Soil properties

Methods used for determining total nitrogen (TN), total phosphorus (TP), available nitrogen (AN), available phosphorus (AP), rapidly available potassium (RP), slowly available potassium (SP), pH, and litter quantity were as previously reported [31]. Briefly, soil pH was measured in a 1:5 soil-to-water suspension using a pH meter. SOC, TN, and TP were determined using the K₂Cr₂O_7_ oxidation method, an elemental analyser, and colorimetry after HClO₄–H₂SO₄ digestion, respectively. AN, AP, and exchangeable base cations were measured following standard extraction and titration or spectrometric methods.

### High-throughput sequencing and analysis

We used amplicon sequencing to identify members of the soil microbial communities. Microbial DNA was extracted by Majorbio (Shanghai, China) using a Fast DNA Spin Kit for soil (MP Biomedicals, USA) according to the manufacturer’s instructions. The V4 region of the 16S rRNA gene, the ITS2 region of nuclear ribosomal DNA, and the V4 region of the 18S rRNA gene were amplified to analyse bacterial, fungal, and protist communities, respectively. Detailed polymerase chain reaction conditions, and data processing procedures are described previously [32], and in the Supplementary Text provided in Note S2.

### Metagenome sequencing and analysis

Metagenomic sequencing was performed on a MiSeq System (Illumina) using the PE150 strategy. Each sample generated ~10 Gb of raw reads. Quality control was performed using Fastp (https://github.com/OpenGene/fastp, v0.20.0) to remove reads <50 bp, with an average quality score of <20, or containing ambiguous bases (N). We used MEGAHIT [33], based on succinct de Bruijn graphs, to assemble and optimize sequences, with contigs ≥300 bp and subjected the contigs to ORF prediction using MetaGene (http://metagene.cb.k.u-tokyo.ac.jp/). Genes with nucleotide lengths ≥100 bp were selected and their sequences translated into amino acid sequences, using Diamond (http://www.diamondsearch.org/index.php, version 0.8.35) to align amino acid sequences of the non-redundant gene set with the KEGG database [34], with BLASTP alignment parameters set to a threshold of 1e-5. Gene abundances associated with the KEGG Ortholog (KO), Pathway, Enzyme, and Module databases were summed to calculate the abundance of their respective functional categories and C metabolism genes were identified by searching KO numbers within the functional annotation results.

The assembled contigs were further processed into metagenome-assembled genomes (MAGs) using metaWRAP v1.3.2 [35] with the metaBat2 [36] binning modules, contigs shorter than 1500 bp were discarded. MAGs were deduplicated using dRep v3.4.3 [37] with the following parameters: a genome completeness of 50% (−comp) and contamination of 10% (−con) by CheckM (v1.0.11) [38], a parallelization factor of 0.9 (−pa), and a species average nucleotide identity (ANI) threshold of 0.95 (−sa). Additionally, the clustering algorithm used was centroid with a larger contig size criterion (−cm), multi-round primary clustering was enabled (−multiround_primary_clustering), and the primary clustering chunk size was set to 5000 (−primary_chunksize). This approach resulted in a non-redundant set of MAGs for subsequent analyses. A total of 1056 representative medium to high quality MAGs (high quality: completeness >90% and contamination <5%; medium-quality >50% and contamination <10%), were classified using GTDB-TK (v.1.6.0) [39] with the “classify_wf” function and default parameters, based on the Genome Taxonomy Database (GTDB) release 202 [40]. GTDB-Tk classifies each genome by calculating ANI and comparing it to a curated set of reference genomes, determining its position within the bacterial or archaeal reference genome tree, and assessing its relative evolutionary distance. The phylogenetic tree was built based on the sequences of 253 MAGs (completeness ≥90% and contamination ≤5%), constructed using GTDB-Tk and visualized with iTOL v7 (https://itol.embl.de/). BLASTN (with an E-value cut-off of 1e-5) was used to identify C metabolism genes. We used BWA v0.7.17 [41] to align the global metagenome clean reads of each metagenome against the nucleic acid sequences of carbon metabolism genes. Low-quality reads were filtered using CoverM v0.6.1, and the abundance of C metabolism genes calculated as reads per kilobase per million mapped reads, based on the number of mapped reads and the lengths of genes.

Carbohydrate-active enzymes (CAZymes) were annotated to investigate microbial functional potential related to organic matter degradation. Protein-coding sequences predicted from metagenomic assemblies were compared against the CAZy database using the dbCAN2 meta server, which integrates HMMER (v3.3), DIAMOND, and Hotpep for improved accuracy [42]. Only CAZyme annotations supported by at least two of the three methods were retained to ensure reliability. Identified CAZymes were classified into functional categories, including glycoside hydrolases (GH), glycosyltransferases (GT), carbohydrate-binding modules (CBM), carbohydrate esterases (CE), polysaccharide lyases (PL), auxiliary activities (AA), and SLH. Relative abundances of CAZyme families were calculated based on normalized gene counts [43].

### Overview of global forest soil metagenomes

We collated 755 metagenomes by searching Web of Science (core database) and the NCBI Sequence Read Archive (https://ncbi.nlm.nih.gov/sra/) database using the keywords “Forest soil microbiome,” “Forest soil metagenome,” “Forest soil macrogenomic,” and ‘Forest soil microorganisms ‘to investigate general patterns of transition associated with microbial carbon fixation functions in planted and natural forests. We applied a standardized protocol for the selection of metagenomic data to minimize bias as follows: (i) Forest plots were not subjected to additional experiments such as inoculation, warming, nutrient alteration and acid deposition; (ii) For those studies that included controlled trials, we only considered the control and excluded the treatment group; (iii) To be included, natural forests had to have been subjected to no anthropogenic impacts (e.g. nature reserves, and managed forests without additional interventions such as the application of fertilizers after planting); (iv) Soil samples must include defined geographic information such as latitude and longitude to enable spatial analysis. Therefore, a total of 755 metagenome datasets from 262 locations were identified. The collated metagenomic datasets including accession number and associated metadata, (habitats, continents, country, and spatial coordinates) are summarized in Supplementary Table S2.

### Determination of soil CO₂ fixation potential

We employed a ^13^CO₂ labeling approach to measure the CO₂ fixation capacity of soils from forests at different successional stages, to further evaluate the CO₂ fixation potential of forest soils [17]. Detailed incubation procedures are provided in Note S3. The soil CO₂ fixation potential was quantified by comparing the ^13^C atomic abundance (atom%) between the labeled and control samples after incubation, with the increase in ^13^C atom% in the labeled sample serving as an indicator of CO₂ fixation capacity. The difference in ^13^C atom% between the labelled and control soils was used to calculate CO₂ fixation potential using the following equation:


$$ \mathrm{Fixation}\ \mathrm{rate}=\frac{\Big({}^{13}{C}_{t\mathrm{reatment}}-{}^{13}{C}_{\mathrm{control}}\Big)\times{C}_{\mathrm{soil}}\times 3.664}{\mathrm{Incubation}\ \mathrm{time}} $$

where fixation rate is the CO₂ fixation potential (mg CO_2_ kg^−1^ soil day^−1^) [13],C_treatment_ is the atom% of treatment sample [13],C_control_ is the atom% of control sample, C_soil_ is the soil carbon content (g·kg^−1^ soil) after the pre-incubation period. 3.664 serves as the conversion factor (from C to CO₂) [44]. Incubation time refers to the total cultivation period (days). Using a light/dark cycle may potentially stimulate some microbial groups that are typically inactive photoautotrophy inactive under natural low-light conditions to express photoautotrophic pathways. Whereas this can provide useful insights into their metabolic potential, it may also lead to a slight overestimation of their actual activity *in situ.* As such, results should be interpreted with caution and, where possible, supported by complementary field-based evidence.

### Network analysis

Microbial co-occurrence networks were constructed using the Molecular Ecological Network Analysis Pipeline (http://ieg4.rccc.ou.edu/mena/) (Table S1). We used the network’s topological structure to study complexity, microbial interactions, and resilience. Number of nodes and links: Represents the total microbial nodes and interaction links, providing an intuitive measure of network interactions. Average degree (average K): Reflects node connectivity. A higher average degree suggests a denser network with more frequent interactions, indicating closer ecological collaboration. Number of keystones: This represents the number of key microorganisms in the network. In the current microbial network analysis, connectors, module hubs, and network hubs represent different node roles. Connectors link multiple modules, facilitating communication between them, while module hubs are central nodes within a specific module that drive internal interactions. Network hubs are highly connected nodes that play a crucial role in interconnecting multiple modules, influencing the overall stability and functionality of the entire network. Average centrality (average CC): Measures a node’s importance in the network. Higher centrality indicates more significant roles within the network. Connectivity (con): Indicates the degree of node interconnection. Higher connectivity suggests stronger interdependence and cooperation among species, whereas lower connectivity points to a sparser structure. RM (average modularity): The microbial network typically consists of multiple modules, with strong interactions within modules and weaker interactions between modules. Reflects the network’s stability. Higher modularity indicates a specialized, adaptable microbial community that can respond to environmental changes whereas maintaining ecosystem health. The vulnerability of microbial networks refers to how disturbances or loss of key species affect ecosystem functionality and stability. The robustness of microbial networks measures their resilience, indicating their ability to recover and maintain ecological functions despite disturbances or environmental changes [45–47].

### Random forest

We used microbial communities and microbial interaction network nodes to predict forest succession stages using a random forest model, to explore whether microbial communities can reflect different stages of forest succession. In this study, we constructed two random forest models, one using the overall microbial community at the ASV level and the other using the network nodes that we constructed. The aim was to determine which of the overall microbial communities or the microbial communities forming the interaction network better represented the different successional stages. We performed 10-fold cross-validation with five repeats to evaluate the importance of the indicators. Once the cross-validation error curve stabilized, the most relevant ASVs were considered microbial indicators for forest succession stages [48].

### Statistics and visualization

Successional differences between soil microbial communities were tested using one-way Analysis of Variance (ANOVA) in SPSS 21 at *P* < 0.05. Microbial communities from the different successional forest types were analysed (Principal Coordinates Analysis, PCoA) and visualized using “vegan” (version 2.5.4) [49] and “ggplot2” (version 3.5.0) [50] R packages, respectively. We used the anosim function from the vegan package in R to calculate the *P* and *r* values for the PCoA. When *P* was much < 0.001, it was marked as *P* < 0.001. To explore microbial community assembly and interactions across forest successional stages, we applied the FEAST [51] algorithm for source tracking and calculated the normalized stochasticity ratio (NST) to assess assembly processes [52]. Network robustness was validated against random networks and visualized using Gephi. Random forest models based on ASVs and network nodes were used to predict successional stages [53]. Finally, a structural equation model was built to quantify the effects of biotic and abiotic factors on microbial network stability and its linkage to carbon fixation potential. For more detailed information regarding the above method, please refer to Supplementary Note S4.

## Results

### Soil microbial communities under forest ecosystem succession

**Figure 1:**
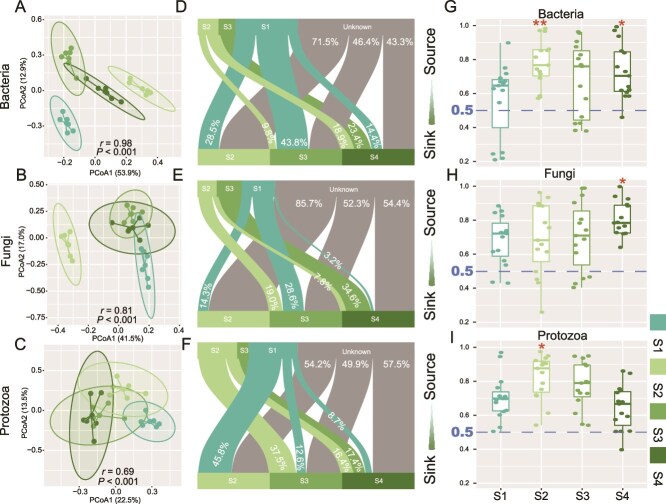
Soil microbial community structure and assembly under subtropical forest succession. Principal coordinates analysis of bacteria (A), fungi (B), and protozoa (C). Fast expectation–maximization microbial source tracking analysis for bacterial (D), fungal (E), and protistan (F) communities. Normalized stochasticity ratio of bacteria (G), fungi (H), and protozoa (I); a NST value >0.5 represents deterministic processes, NST < 0.5 represents stochastic processes. S1: Pine forest; S2: Mixed pine-broadleaf; S3: Mixed pine-broadleaf with elder; S4: Monsoon evergreen-broadleaf.

Composition of the soil bacterial community differed at each forest successional stage (*r* = 0.98*, P* < 0.001), separating primarily along the PC1 axis (Fig. 1A). Although less distinct, fungal (*r* = 0.81*, P* < 0.001) and protistan communities (*r* = 0.69*, P* < 0.001) also differed with succession (Fig. 1B and C). The difference in fungal communities was likely driven by the S2 fungal communities; and for protozoa, differences were likely driven with communities associated with S1 (Fig. 1B and C).

Using FEAST for microbial source tracking analysis, it was found that S1, S2, and S3 collectively contributed 56.7% of the bacterial community, 45.6% of the fungal community, and 42.5% of the protistan community at the later stages of succession (Fig. 1D-F). Whereas the alpha diversity of the bacterial communities did not change (*P* > 0.05) with forest successional stage, the alpha diversity of soil fungi increased with successional stage (*P* < 0.05). Protozoa associated with the most mature forest successional stage (S4) was significantly greater than the other successional stages (Fig. S1).

For bacteria at the phylum level, members of *Proteobacteria*, *Actinobacteriota*, *Acidobacteriota*, *WPS-2*, and *Chloroflexi* were dominant across all forest successional stages. For bacteria at the family level, members of *Xanthobacteraceae*, *Acidothermaceae*, *Acetobacteraceae*, *Acidobacteriaceae*, and *Solirubrobacteraceae* were the five most abundant. For fungi, the relative abundance of members of the *Mortierellomycota*, *Basidiomycota*, and *Ascomycota* phyla accounted for >75% of the fungal community across all successional stages, with the highest relative abundance associated with *Mortierellaceae* members. The relative abundance of the protistan *Apicomplexa* phylum was significantly higher in S4 compared to S1, whereas the abundance of *Enchytraeidae* members decreased (*P* < 0.05) (Fig. S2).

At all stages of forest succession, the assembly processes of bacterial, fungal, and protist communities were predominantly driven by stochastic processes (Fig. 1G-I). The assembly of bacterial and fungal communities increasingly became influenced by stochastic processes as succession progressed (Fig. 1G and H). In other words, compared to the later stages of succession, bacterial and fungal communities in the early stages of succession were more influenced by environmental pressures in their community assembly. In addition, the protist community was most strongly dominated by stochastic processes at S2, after which it gradually became more influenced by deterministic processes (Fig. 1I).

### Soil physicochemical properties and forest richness

Soil physicochemical properties were highly variable across the forest successional stages. Soil organic carbon (SOC), TN, and litter biomass increased significantly with successional stage, with SOC in S4 being 1.388 times higher than in S1. Moreover, pH was statistically greater in the mature forest successional stage. The forest canopy closure was 70% (S1), 90% (S2), 90% (S3), and 95% (S4), respectively (Fig. S3A).

Tree richness significantly increased with forest succession (Fig. S3B). In contrast, the understory exhibited a more variable pattern that did not align with successional stage. S3 had the lowest understory species richness, whereas the species richness of the understory vegetation associated with S4 and S2 was significantly greater than either of the other two successional stages, S1 and S3 (Fig. S3B).

### Succession and underlying mechanisms of microbial networks

**Figure 2:**
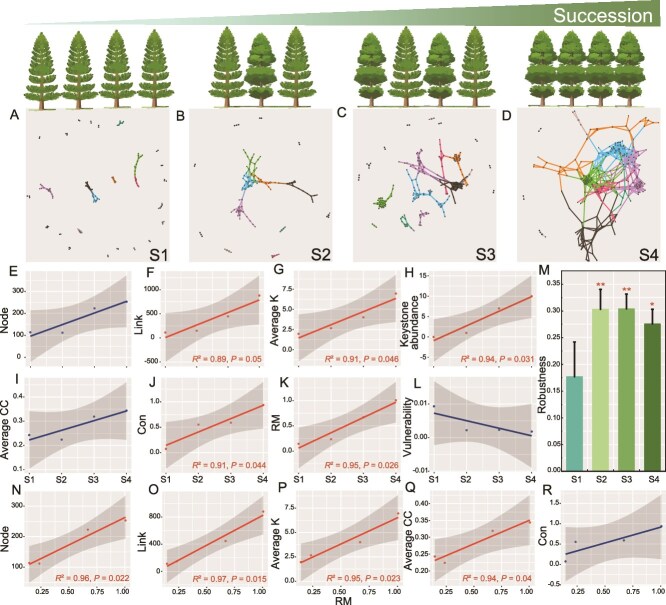
Soil microbial networks under subtropical forest succession. Visualization of microbial networks under forest succession (A-D) with modules containing more than 5 nodes colored. Changes in network topology structure across forest successional stages, including network node (E), network link (F), average degrees (average K) (G), number of keystones (H), average CC (I), connectivity (con) (J), relative modularity (RM) (K), vulnerability (I) and robustness (M). Correlation between relative modularity and network topological characteristics (N-R). (^*^P ≤ 0.05，^**^P ≤ 0.01). S1: Pine forest; S2: Mixed pine-broadleaf; S3: Mixed pine-broadleaf with elder; S4: Monsoon evergreen-broadleaf.

Forest soil microbial communities became increasingly complex during forest succession (Fig. 2A-D) (Table S2). The number of network links (*R*^2^ = 0.89*, P* = 0.05), average degrees (Average K) (*R*^2^ = 0*.*91*, P* = 0.046), the number of keystones (*R*^2^ = 0.94*, P* = 0.031), connectivity (Con) (*R*^2^ = 0.91*, P* = 0.044) and relative modularity (RM) (*R*^2^ = 0.95*, P* = 0.026) increased during forest succession (Fig. 2F, G, H, J, K). The robustness of the microbial network structure was lowest at the S1 stage, which represents the least mature stage of forest succession; the soil microbial network robustness of multi-species forests (S2, S3, and S4) was 67.4% higher than that of single-species (S1) forests (Fig. 2M). Relative modularity of the microbial communities increased during forest succession with an increase of network nodes (*R*^2^ = 0.96*, P* = 0.022), links (*R*^2^ = 0.97*, P* = 0.015), average degrees (*R*^2^ = 0.95*, P* = 0.023), and average clustering coefficient (Average CC) (*R*^2^ = 0.92*, P* = 0.04) (Fig. 2N-Q). The results of the number of network links, average K, connectivity, and RM indicated that microbial interaction relationships in the microbial network significantly strengthened with forest succession. Meanwhile, the results of the number of keystones and robustness suggested that the structure of the microbial interaction network became more complex in the later stages of forest succession, with increased resilience. The ratio of positive to negative network connections varied with successional stage, with positive connections representing 87.5% of the total network connections in S1, whereas in S4, the proportion of positive and negative connections approached parity (54.5:45.5%), suggesting that microbial interaction relationships tend to balance in the later stages of forest succession, which also implies that the interaction network becomes more stable (Fig. S4A). For S2, S3, and S4, a total of 1, 7, and 7 module hubs were identified, with an additional three network connectors found in the S4 treatment (Fig. S4B). The number of unique network nodes increased with forest succession (Fig. S4C).

**Figure 3:**
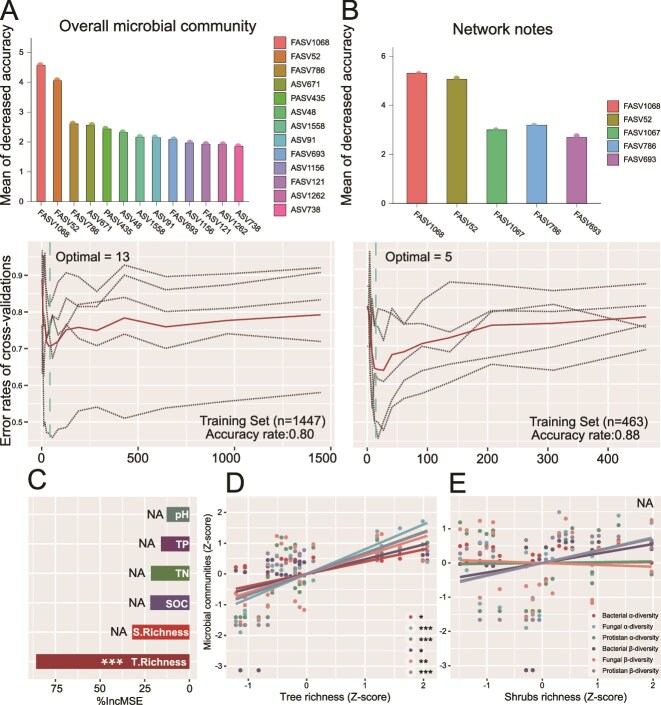
Random forest evaluation of the representatives of microbial communities and the ranking of the importance of forest ecosystem indications. Random forest model for the identification of indicator species for different forest types, using the overall microbial community (A), and network nodes (B) separately. The relative importance of different factors in influencing microbial community composition under succession (C), linear regression of tree (D), and shrub (E) diversity with the communities of bacteria, fungi, and protists. The microbial community data were standardized using Z-scores (^*^P ≤ 0.05，^**^P ≤ 0.01，^***^P ≤ 0.001). T. Richness and S. Richness represent the richness of tree and shrub species, respectively. SOC: Soil organic carbon; TN: Total nitrogen; TP: Total phosphorus; NA: no significance.

We constructed random forest models based on microbial ASVs to distinguish microbial community differences across successional stages and to classify forest successional status, thereby supporting Hypothesis 1. The machine learning results showed that when using microbial community structure at the ASV level for random forest modelling, 13 ASVs were identified as key indicators of forest successional differences. However, when modelling with microbial network nodes, 5 ASVs were defined as key indicators, and the prediction accuracy for succession status was higher (88% vs 80%) than that of the previous model, indicating that the microbial interaction network we constructed is not randomly composed and can effectively represent different stages of forest succession (Fig. 3A and B). Ranking the importance of forest ecosystem indicators across the different successional stages showed that tree richness was the primary driver of forest succession in the system under study (*P* < 0.001) (Fig. 3C). As a result, we conducted separate analyses to evaluate correlations between tree richness, shrub richness and alpha and beta diversity of bacteria, fungi, and protozoa, respectively. Tree richness (Fig. 3D) had positive correlations of differing levels of statistical significance with both alpha and beta diversity for all microbial communities, whereas shrub richness (Fig. 3E) exhibited no relationship.

### Microbial functional potential changes with forest succession

The primary function associated with the three microbial groups was metabolism, with a lesser functional contribution associated with organismal systems, cellular processes, and environmental information processing (Fig. S5A). In the early stages of succession (S1 and S2), the abundance of genes related to metabolism and genetic information processing was greater compared to the later successional stages (S3 and S4). Conversely, the abundance of genes related to microbial “environmental information processing”, including membrane transport, signal transduction and signaling molecules and interaction, was significantly greater in the later (S3 and S4) compared to the earlier forest succession stages (Fig. S5 B-F). Overall, microbial function diverged with forest successional stage (*r* = 0.84*, P* < 0.001) (Fig. S6A). A total of 3110 metabolically related genes were detected from across all four forest successional stages, of which 666 genes were enriched in S1 and 291 in S4 (Fig. S6B) with the remainder (*n* = 2153) having no preference for any particular successional stage.

**Figure 4:**
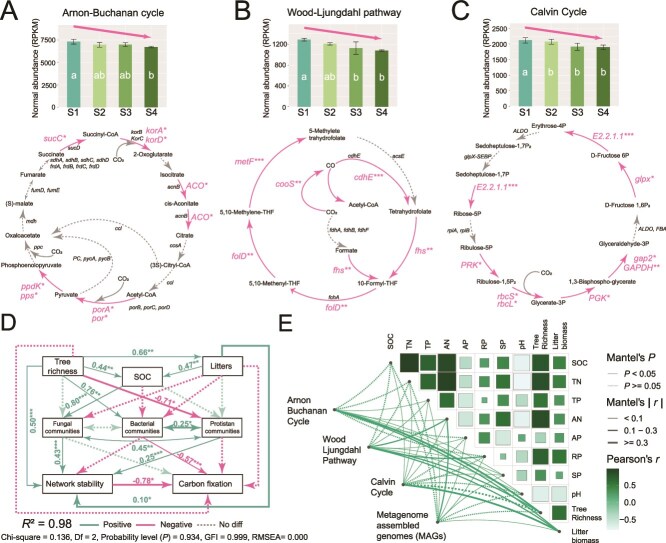
Successional effects on soil microbial functional potential. Carbon fixation pathways include the Arnon–Buchanan cycle (A), wood–Ljungdahl pathway (B), and Calvin cycle (C). Genes marked with significance on each pathway diagram indicate a significant increase, decrease, or no difference in abundance in the later stages of forest succession compared to the earlier stages. Structural equation model (χ^2^ = 0.136, *P* = 0.934, df = 2, GFI = 0.999, RMSEA = 0.000) highlighting impacts of soil properties, plant richness, litter biomass, and microbial communities (bacteria, fungi, protists) on network stability and carbon fixation (D). Positive and negative correlations respectively indicate whether factors increase or decrease network stability; correlation heatmap linking carbon fixation, microbial functions, and ecosystem indicators (E). TN: Total nitrogen; TP: Total phosphorus; AN: Available nitrogen; AP: Available phosphorus; RP: Rapidly available potassium; SP: Slowly available potassium. S1: Pine forest; S2: Mixed pine-broadleaf; S3: Mixed pine-broadleaf with elder; S4: Monsoon evergreen-broadleaf. (^*^P ≤ 0.05，^**^P ≤ 0.01，^***^P ≤ 0.001).

Whereas carbon metabolism related functions associated with S3 were slightly elevated (*P* < 0.05), there was no trend with successional stage (Fig. S7). However, the RPKM abundance of carbon fixation pathways, including carbon fixation in prokaryotes (ko00720) and carbon fixation in photosynthetic organisms (ko00710), generally decreased during forest succession (Fig. S8A and B), with S1 being 11.0% and 9.8% higher than S4 in the ko00710 and ko00720 pathways, respectively. In particular, the abundance of the reductive citrate cycle (Arnon–Buchanan cycle), reductive acetyl-CoA pathway (Wood–Ljundahl pathway), reductive pentose phosphate cycle (Calvin cycle) (Fig. 4A-C), hydroxy propionate-hydroxybutyrate cycle, dicarboxylate-hydroxybutyrate cycle, light crassulacean acid metabolism (CAM) and C4-dicarboxylic acid cycle NAD-malic enzyme type (Fig. S8B) generally decreased under forest succession. During succession, there was a significant decrease in abundance of *sucC*, *korA*, *ACO*, *porA*, *por*, *ppdK* and *pps* genes associated with the Arnon-Buchanan cycle (Fig. 4A). In the Wood-Ljungdahl pathway, 7 of the 13 processes exhibited significant decline (Fig. 4B). Similarly, 9 of the 15 genes related to the Calvin cycle significantly decreased (Fig. 4C). Except for S3, there were few differences in pathways related to nitrogen metabolism with only genes related to nitrification differing between successional extremes (Fig. S8C).

We used the CAZymes database to annotate carbohydrate-active enzyme (Fig. S9). Our analysis revealed no differences in overall CAZymes abundance during forest succession. However, distinct classes exhibited significant changes in the later stages of succession. For instance, the abundances of CBM, GH, and SLH increased significantly with succession (*P* < 0.05) (Fig. S9A). Further analysis of individual CAZymes families identified 79 families whose abundances were significantly positively correlated with forest succession. These successional-associated families were predominantly from the GH class, accounting for 51 families, which represent 64.6% of the total (Fig. S9B).

Structural equation model suggested putative effects, indicating that forest tree richness, litter biomass, and fungal and protistan community structure may have been positively associated with microbial network stability, potentially explaining 98% of the variance (Fig. 4d). Bacterial community structure and microbial network stability might have been negatively associated with the abundance of carbon fixation potential genes (Fig. 4d). Forest tree richness (*λ* = 0.50, *P* < 0.001) was the main driver of microbial network stability, possibly regulating bacterial (*λ* = 0.76, *P* = 0.005) and protistan (*λ* = −0.71, *P* = 0.013) communities, which may have indirectly influenced network stability. Conversely, factors such as tree richness, litter biomass, and fungal and protistan communities, rather than bacterial communities, were found to exhibit significant positive associations with carbon fixation potential (Fig. S10a). Tree richness was the most important factor in promoting the stability of microbial networks and causing a reduction in the abundance of microbial C fixation function (Figs. S10a, b). The Arnon-Buchanan cycle, Wood-Ljungdahl pathway, and Calvin cycle significantly correlated with litter biomass in the different forest successions. Additionally, tree richness influenced the Arnon-Buchanan cycle and Wood-Ljungdahl pathway. The composition of MAGs was correlated with soil AN (Fig. 4E).

Based on our metagenomic data, a total of 26 (22 bacterial, 4 archaeal) high-quality metagenome-assembled genomes (MAGs) were constructed and dominated by *Acidobacteriota* and *Pseudomonadota* members (Fig. S11A). The composition and structure of bacterial MAGs diverged across the successional stages (*r* = 0.87*, P* < 0.001), with the two later successional stages, S3 and S4, being more similar to each other than either of the earliest successional stages (S1 and S2) (Fig. S11b). The relative abundances of MAG4, MAG5, MAG20, MAG26, MAG23, MAG12, and MAG13 were greatest in the early successional stage (S1), whereas MAG3, MAG14, and MAG19 had the greatest relative abundance associated with the most mature successional stage, S4 (Fig. S12a). Furthermore, we calculated the abundance of carbon fixation pathways (ko00720 and ko00710) based on the gene abundances carried by the MAGs we obtained. We found that these two functional pathways also showed a decreasing trend in MAGs as forest succession progressed (Fig. S12b). The relative abundance of the bacterial families *Acetobacteraceae*, *Pseudonocardiaceae*, and *Treboniaceae* members were positively correlated with both the ko00720 and ko00710 pathways, whereas the relative abundance of *Nitrobacteraceae* and *Mycobacteriaceae* members were negatively correlated (Fig. S12c).

**Figure 5:**
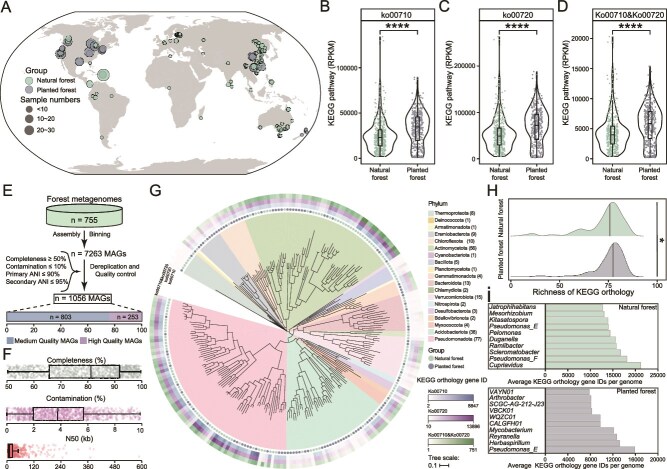
Global patterns of forest soil microbial carbon fixation. Geographic distribution of collated forest soil metagenomes (A). Microbial carbon fixation in prokaryotes (B), carbon fixation in photosynthetic organisms (C) and both carbon fixation pathways combined (D), across global natural and planted forests. A total of 7263 MAGs were recovered from collated global metagenomes of planted and natural forests (E). All MAGs were ≥ 50% complete, were ≤ 10% contaminated. Distribution of quality metrics across the MAGs (F). The maximum-likelihood tree of 253 MAGs. Clades are colored according to the source of genomes. The outer heatmaps, based on the number of annotated gene IDs, indicate whether each genome possesses the potential for carbon fixation via ko00710, ko00720, or both pathways. (G). Differences in the richness of carbon fixation homolog genes carried between each genome in planted and natural forests (H). The top 10 genus carrying the most carbon fixation homolog genes in planted and natural forests (I).

We collated 755 metagenomic datasets representing forest soils (both natural and managed) from 24 countries, to further validate the MAG results from our experimental comparisons (Fig. 5A). By comparing the ko00720 and ko00710 pathways in the KEGG database, we found that the functional abundances of carbon fixation in prokaryotes (Fig. 5B) and carbon fixation in photosynthetic organisms (Fig. 5C) were significantly higher in the managed forest samples than in the natural forest samples, with the abundance of Ko00710 in the planted forest being 1.31 times that of the natural forest, whereas Ko00720 was 1.30 times higher. Furthermore, we recruited 7263 genomes from 755 metagenomes. After quality evaluation and deduplication, we obtained 1056 medium to high-quality (> 50% completeness and < 10% contamination) genomes (Figs. 5 E, F). We retained 253 high-quality genomes (> 90% completeness and < 5% contamination) for further analysis, of which 142 were from natural forests, and 111 were from plantations (Supplementary Table S3). Most of the 253 genomes belonged to bacteria, with 8 taxa belonging to the Archaea phylum, *Thermoproteota*. For genomic annotation analysis, a total of 108 carbon fixation-related KEGG orthologs were detected in 253 genomes, with at least one gene associated with carbon fixation present in each genome (Supplementary Table S4). The taxonomic assignment revealed that 77 and 56 genomes were assigned to the top two phyla, *Pseudomonadota* and *Actinomycota* members, respectively, followed by 38 genomes for *Acidobacteriota* members (Fig. 5G). After evaluating the carbon fixation potential of these genomes, we found that members of *Pseudomonadota* and *Actinomycota* carried a rich abundance of carbon fixation genes related to ko00710 and ko00720 (Fig. 5G). It suggests that members of *Pseudomonadota* and *Actinomycota* are the main microbial phylum involved in carbon fixation in forest ecosystems. We further compared the differences in the types of KEGG homologous genes carried in the local genomes of planted and natural forests, and found that the planted forest genomes carried more carbon sequestration genes, which may also contribute to the differences in carbon sequestration between planted and natural forests (Fig. 5H). The carbon fixation community of planted and natural forests differed significantly at the genus level, with planted forests dominated by *Pseudomonas* and *Herbaspirillum* members whereas natural forests were dominated by *Cupriavidus* and *Pseudomonas* members (Fig. 5I).

**Figure 6:**
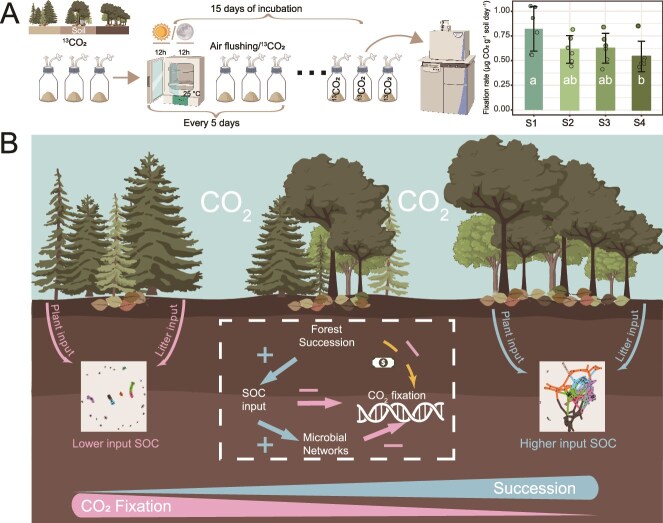
Incubation of soil under ^13^CO_2_ labeling conditions. Outline diagram illustrating soil incubation under ^13^CO₂ labeling conditions and the fixation rate of CO₂ by successional stage (A). Overview of mechanisms influencing soil microbial CO_2_ fixation function (B). S1: Pine forest; S2: Mixed pine-broadleaf; S3: Mixed pine-broadleaf with elder; S4: Monsoon evergreen-broadleaf.

We conducted a ^13^C-labelling incubation experiment (Fig. 6A) to further evaluate the microbial CO₂ fixation rate, which showed that soil CO₂ fixation rates ranged from 0.41 to 1.05 μg CO₂ g^−1^ soil day^−1^, with the highest rate observed in S1 and the lowest rate found in S4. Overall, the CO₂ fixation rate exhibited a decreasing trend along the forest succession gradient.

## Discussion

Microbial communities were significantly correlated with tree diversity and reflect different stages of forest succession, supporting our first hypothesis. Moreover, an increase in tree diversity with forest succession promoted the complexity and resilience of microbial networks, consistent with our second hypothesis. Carbon fixation functional potential declined at plot scale, which, when combined with global and ^13^C labelling data, revealed that as SOC increased, the role of autotrophic microorganisms likely decreased, leading to a reduction in the relative abundance of carbon fixation genes, supporting hypothesis 3 (Fig. 6B). Although this study found a decline in microbial carbon fixation along forest succession, the study design and metagenomic analysis had certain limitations. Firstly, as metagenomics unable to distinguish between live and dormant microorganisms, genomic abundance cannot be accurately estimated, which may lead to either an overestimation or underestimation of microbial carbon fixation potential. Secondly, as laboratory experiments inevitably cannot fully replicate field conditions, there may be an overestimation of microbial activity *in situ*. Therefore, future work will need to integrate multiple dimensions and methodologies to accurately assess the contribution of soil microbial carbon fixation to the overall carbon sequestration potential of ecosystems.

### Successional shifts in forest soil microbial community and networks

Tree richness and soil nutrient levels increased with succession, with a concomitant more complex and resilient microbial interaction network, suggesting synchronous succession of plant and soil microbial communities in the studied subtropical forest system. This concurs with previous reports that biological communities become more complex under succession [54, 55]. In this study, tree richness, the most representative indicator of forest ecosystem succession, was positively correlated with microbial diversity indices, likely driven by the release of root exudates that enhance SOC and subsequently recruit and facilitate microbial colonization and associated interactions [56, 57]. The results of the structural equation model confirmed our hypothesis that aboveground vegetation diversity significantly and synchronously promotes community β-diversity of soil bacteria, fungi, and protists. Although we found no successional differences in bacterial α-diversity (Shannon Index), there were successional differences in microbial community β-diversity (composition), possibly due to shifts in competitive advantage of recruited microbial taxa [58, 59] as a result of increased soil nutrient content leading to stochastic drivers of microbial community assembly [60]. Similarly, changes in microbial networks are known to reflect the adaptive processes of microbial communities to environmental changes [61, 62]. Our results support this finding, showing that network links, average K, number of keystones, connectivity, relative modularity, and robustness significantly increase, indicating that microbial networks become increasingly complex and more stable with succession [45]. Similar to effects of climate warming on microbial networks, this study highlights successional changes in subtropical forest soil microbial networks that then likely enhance microbial interactions [63]. The S4 microbial network had close to parity (54.46%) in terms of positive and negative correlations, whereas in the S1 microbial network, positive correlations dominated (87.5%). The balance between symbiosis (positive correlations) and competition (negative correlations) in the microbial interaction network may help enhance the adaptability and disturbance resistance of the microbial community, thereby improving the resilience of the ecosystem [64]. However, for the early stages of succession, a higher degree of symbiosis may lead to metabolic dependencies. Thus, in S1, more metabolic functional redundancy is required to maintain the interrelationships among microorganisms [64]. The composition of bacterial, fungal, and protistan communities influenced network stability, with fungi eliciting the most pronounced impact, possibly as a result of shifts in dominant taxa from early to late stages of succession [65]. Furthermore, fast growing bacterial communities with high species turnover dominated in early successional stages, whereas fungi become predominant in later stages [66]. As suggested [67], despite competition among different fungi for resources and habitats, as forest succession progresses, diverse tree species provide sufficient resource and habitat diversity to reduce competition. Diverse types of litter significantly promote fungal diversity, altering composition of soil fungal community and significantly increasing the positive effects of complementary resource utilization [68].

### Late succession impacts microbial functional potential

Genes associated with microbial metabolism related functions accounted for the largest proportion of functional categories and their potential was greatest in the earlier stages of subtropical forest succession. This may reflect that microorganisms have a greater functional potential under early succession to adapt to relatively nutrient-limited soil conditions [69]. Microbial carbon fixation functional genes can characterize the soil carbon sequestration process in which microorganisms participate [70]. Data from our ^13^C labelling incubation experiment showed that the carbon fixation rate in S4 was significantly lower than in S1. Coupled with the data on carbon fixation functional genes, suggests that the decline in functional potential was synchronous with the reduction in actual microbial carbon fixation activity in soil. This decline is likely driven by the quantity and quality of available SOC [71]. Our previous study suggests that in the early stages of succession, due to the lower diversity of the vegetation community in the early stages of succession, the diversity of litter is low, leading to a limited source of soil organic carbon. However, due to the lower quality of litter in the early stages of succession (higher C/N ratio) compared to the later stages, the proportion of litter decomposition products transferred to the soil is low, thereby affecting the carbon supply to soil microorganisms [72]. The activity of GHs reflects the potential for polysaccharide decomposition, such as that of cellulose, hemicellulose, and starch, as these enzymes break down complex carbohydrates into simpler sugars for microbial utilization [73]. The significant increase in GH activity during the later stages of succession, compared to the early stages, may indicate an accelerated decomposition of organic carbon by microorganisms, which is due to significant differences in microbial communities between mixed and single-species litter, with polysaccharide-degrading enzymes being significantly higher in mixed litter than in pine forest samples [74]. Under field conditions, the dominant bacterial genera *Duganella* and *Cupriavidus* members were more involved in carbon decomposition processes in natural forests. Previous studies have reported that *Duganella* sp. possesses cellulose-degrading capabilities, whereas the members of genus *Cupriavidus* actively participate in carbon dynamics [75, 76]. As a result, this promotes litter decomposition, which in turn enhances soil quality and microbial carbon utilization efficiency [77]. This suggests that in the later stages of succession, microbial communities shift from directly fixing carbon dioxide to relying on soil organic matter.

Lower soil nutrient content results in the selection and enrichment of microorganisms with higher carbon fixation potential for survival; however, as succession progresses, above-ground plant diversity increases [78], along with greater diversity and complexity in plant root systems and root exudates [79]. Simultaneously, there is diversification in soil SOC sources, including litter decomposition and plant root transport, which transfers organic carbon from above-ground to below-ground [80, 81], recruiting different microorganisms. Furthermore, we found that the functional potential for nitrification was significantly higher in the later stages of succession compared to the earlier stages, suggesting that increased nitrogen availability may promote plant growth and lead to more SOC being transferred from above-ground to below-ground. However, this hypothesis requires further validation. Our results showed a significant increase in forest canopy closure as forest succession progressed. This could affect soil microbial enzyme activity [82]. In contrast, as photosynthetic microorganisms, such as cyanobacteria, were present in the surface soil [83], it was hypothesized that with increasing canopy cover, the abundance of these microorganisms likely decreased, which could, in turn, reduce carbon fixation potential in the later stages of forest canopy development. Our results indicated that the relative abundance of the light CAM pathway significantly decreased with succession, supporting that greater forest canopy closure was also one of the factors influencing soil microbial carbon dioxide fixation functions. Hence, in the context of unchanged bacterial α- diversity, shifts in relative abundances among different taxa within bacterial communities may be an important driver of variations in relative abundance of microbial carbon fixation genes, which is consistent with other relevant studies on the relationship between microbial communities and functional genes [84–86]. A similar pattern was evident in the MAGs we compared in field, where the proportion of carbon-fixing microorganisms was greater at the early stages of succession. Thus, we consider that under successional processes, microbial carbon fixation is not the primary mechanism for carbon sequestration in subtropical forest ecosystems, despite increasing diversity and enhanced interactions among soil microbial communities and networks. However, for plantation forests or secondary forests with low organic carbon content, microbial carbon fixation may contribute to SOC storage and soil fertility enhancement. When comparing global forest soil metagenome data, we found that, for natural forests, soil microbial carbon fixation potential in planted forests was significantly higher, which strongly supports this viewpoint. However, the actual microbial carbon fixation in plantation forests versus natural forests requires further research. Conversely, in resource-limited environments, microorganisms likely employ multiple pathways to secure essential nutrients to enable adaption to environmental fluctuations and resource scarcity. However, as SOC accumulates, taxa containing these carbon fixation pathways are replaced or selected, resulting in a reduced level of carbon fixation ability [87].

## Conclusion

We observed synchronized microbial community succession alongside forest succession in a subtropical forest system. Furthermore, microbial networks exhibited increased complexity and resilience throughout forest succession. Soil microbial functional potential varied between late and early successional stages, suggesting a reduced functional potential related to metabolism in the later stages. Soil microbial carbon fixation potential showed a significant decline with forest succession. This trend was further supported by a global dataset of metagenomes, which revealed that microbial carbon fixation potential was higher in planted forests compared to natural forests. Results from ^13^C labelling experiments corroborated these findings, showing a clear reduction in carbon fixation rates as succession progressed. Overall, this study deepens our understanding of the future potential of soil microorganisms in ecosystem carbon cycling processes and contributes to the accurate prediction of forest ecosystem carbon cycling dynamics.

## Supplementary Material

Figure_S1_wraf191

Figure_S2_wraf191

Figure_S3_wraf191

Figure_S5_wraf191

Figure_S6_wraf191

Figure_S7_wraf191

Figure_S8_wraf191

Figure_S9_wraf191

Figure_S9_wraf191

Figure_S10_wraf191

Figure_S11_wraf191

Figure_S12_wraf191

Supplementary_materials_wraf191

Supplement_Table_2_wraf191

Supplementary_Table_3_wraf191

Supplementary_Table_4_wraf191

## Data Availability

The data underlying this article are available in National Center for Biotechnology Information (NCBI), and can be accessed with accession numbers PRJNA1031415 (bacterial), PRJNA1031425 (fungal), PRJNA1031708 (protistan) and PRJNA1032280 (metagenomic).

## Classification

Ecology.
