# Supplementary figures and images for: Multi-scale evidence for declining microbial carbon fixation along forest succession gradients
Source: ISME J. 2025 Aug 24;19(1):wraf191. doi: 10.1093/ismejo/wraf191 (PMC12448444; doi:10.1093/ismejo/wraf191)

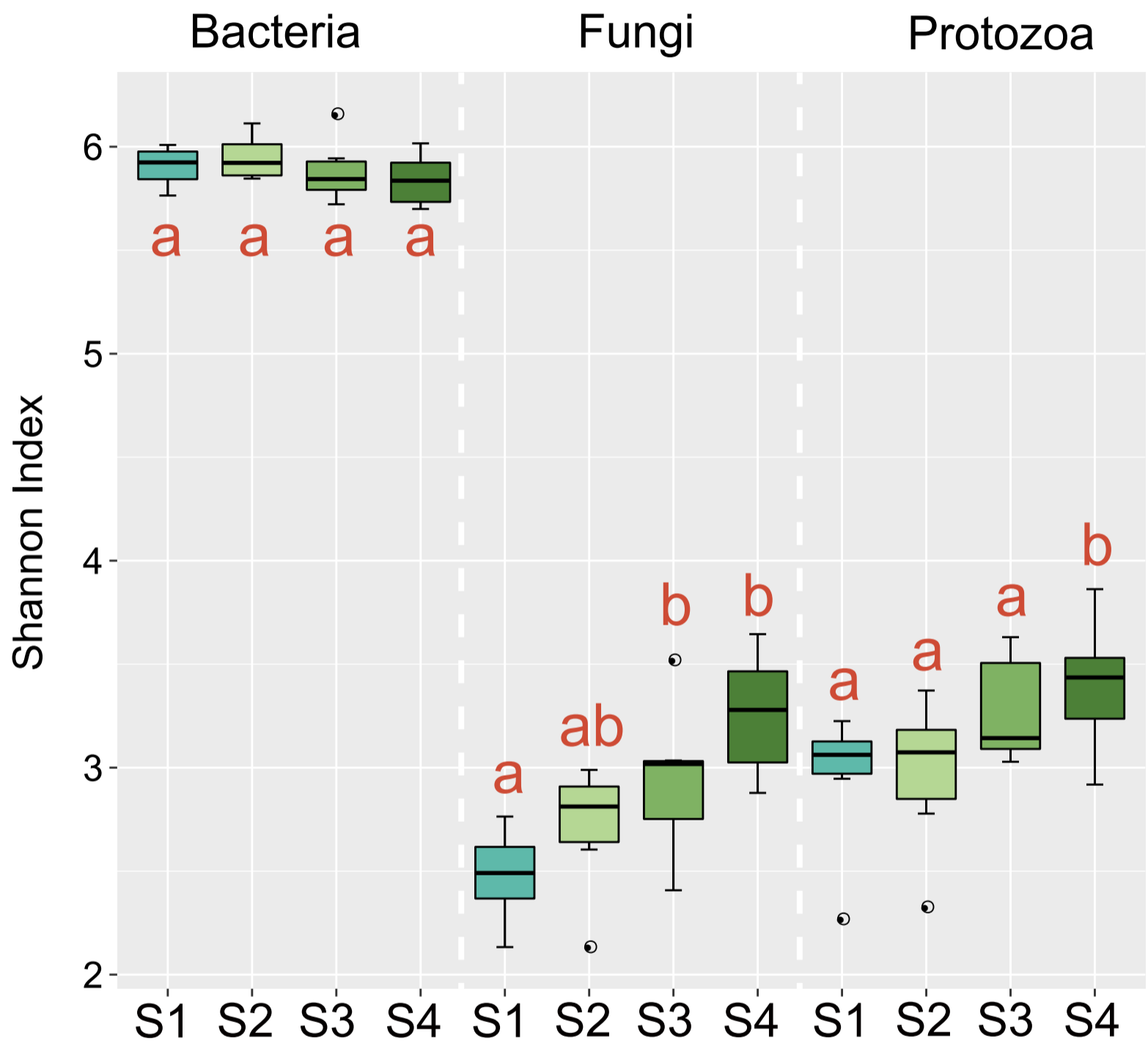

Supplement: Figure_S1_wraf191 [file figure_s1_wraf191.pdf]

# a Bacterial

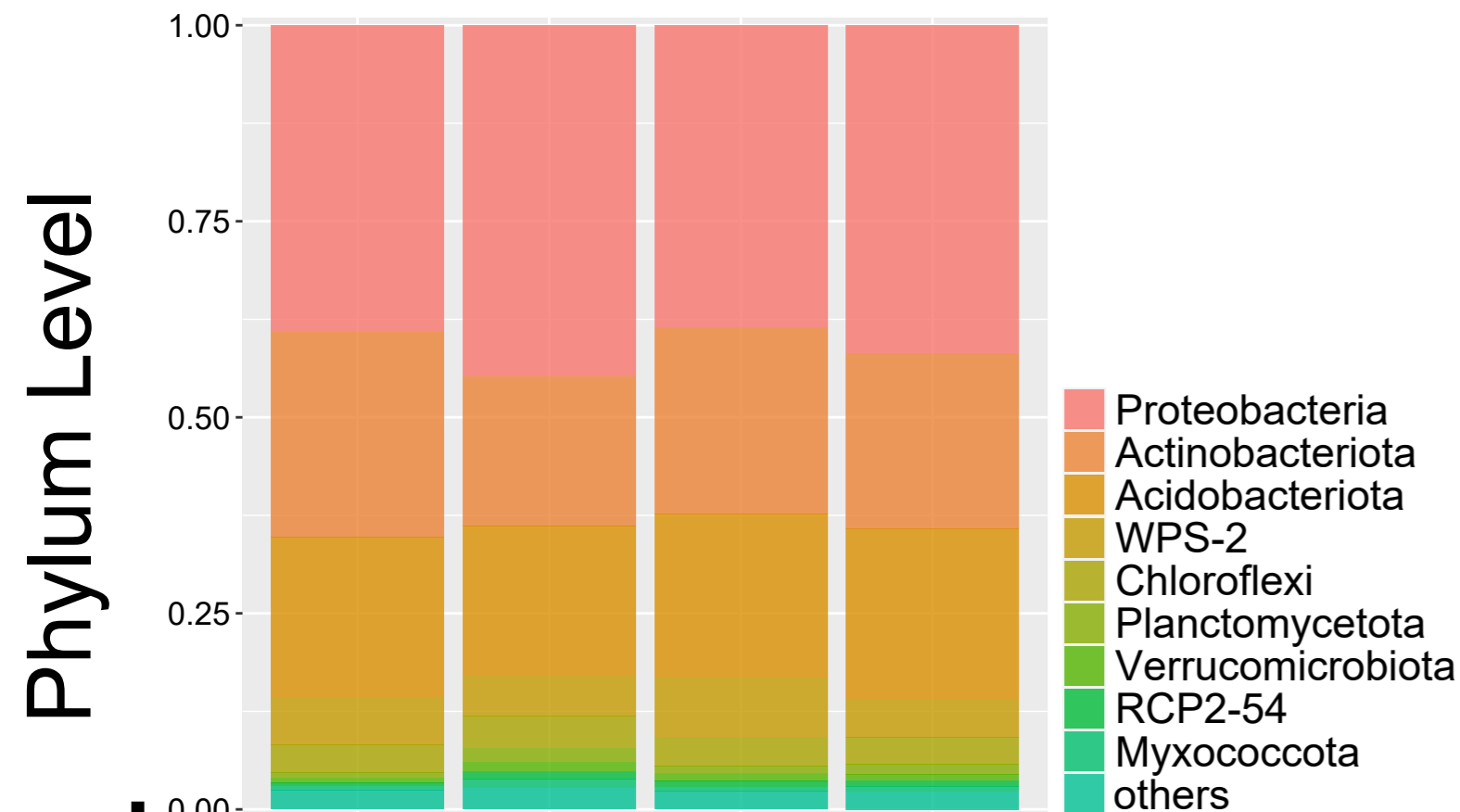

# b Fungal

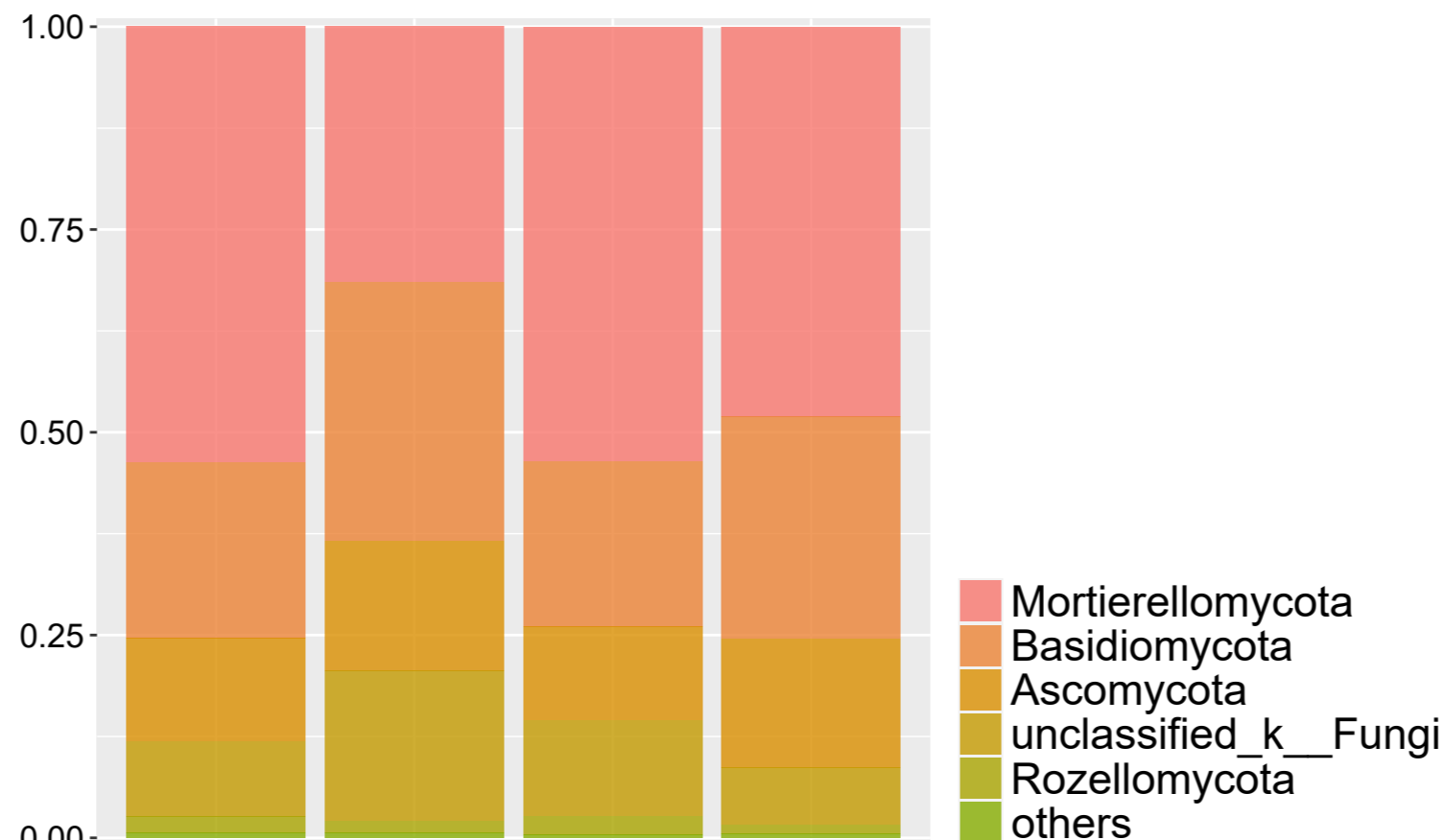

# c Protistan

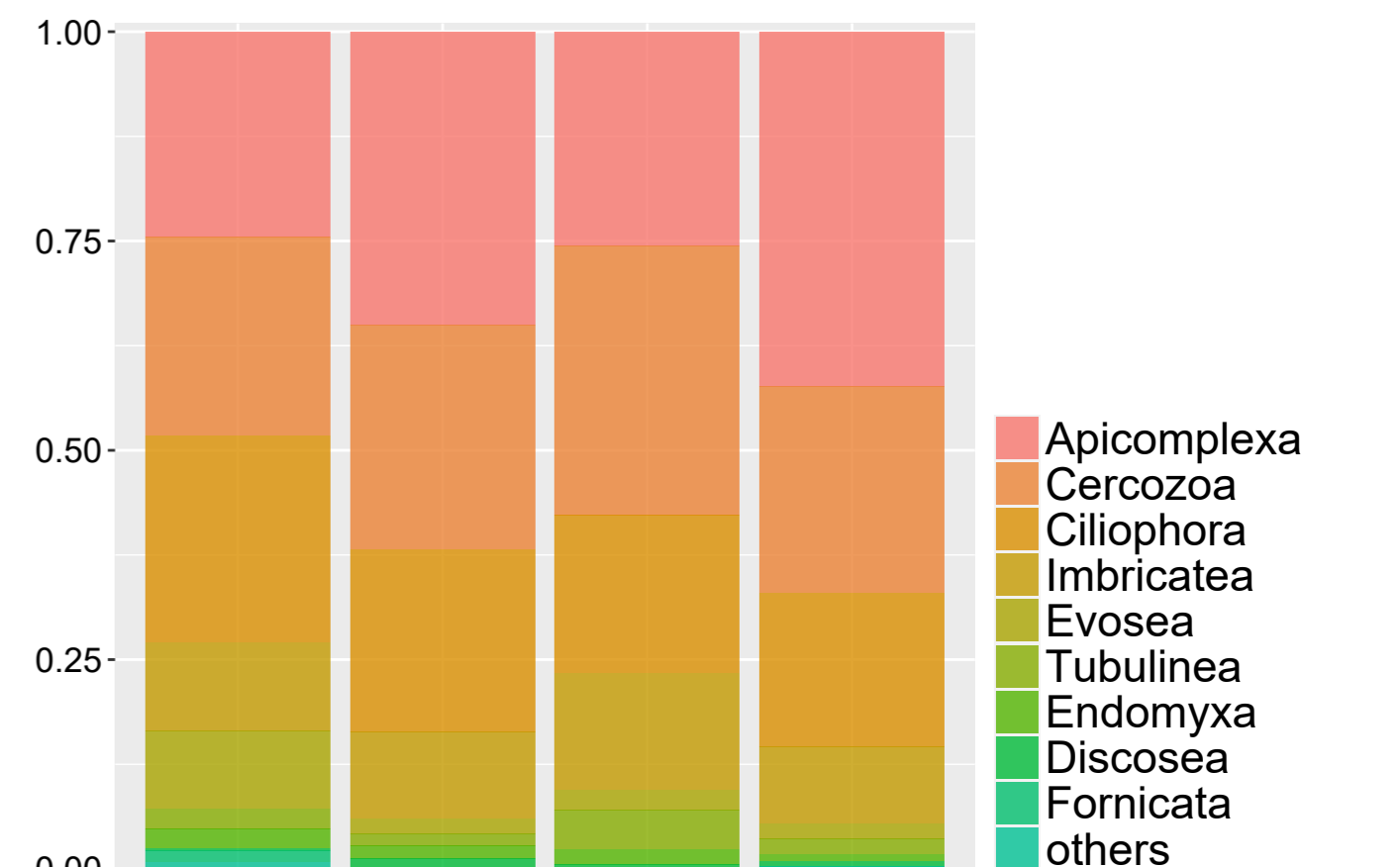

# d

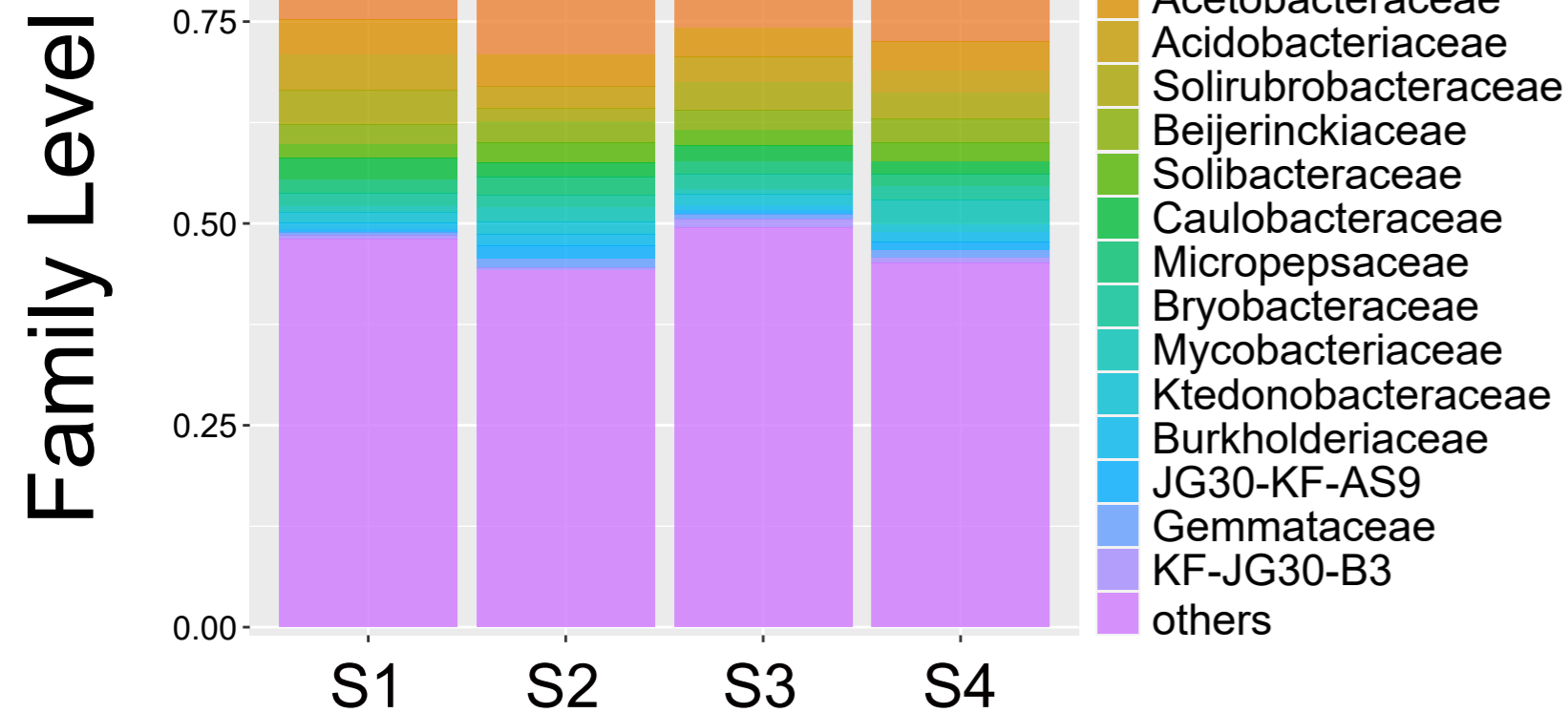

# e

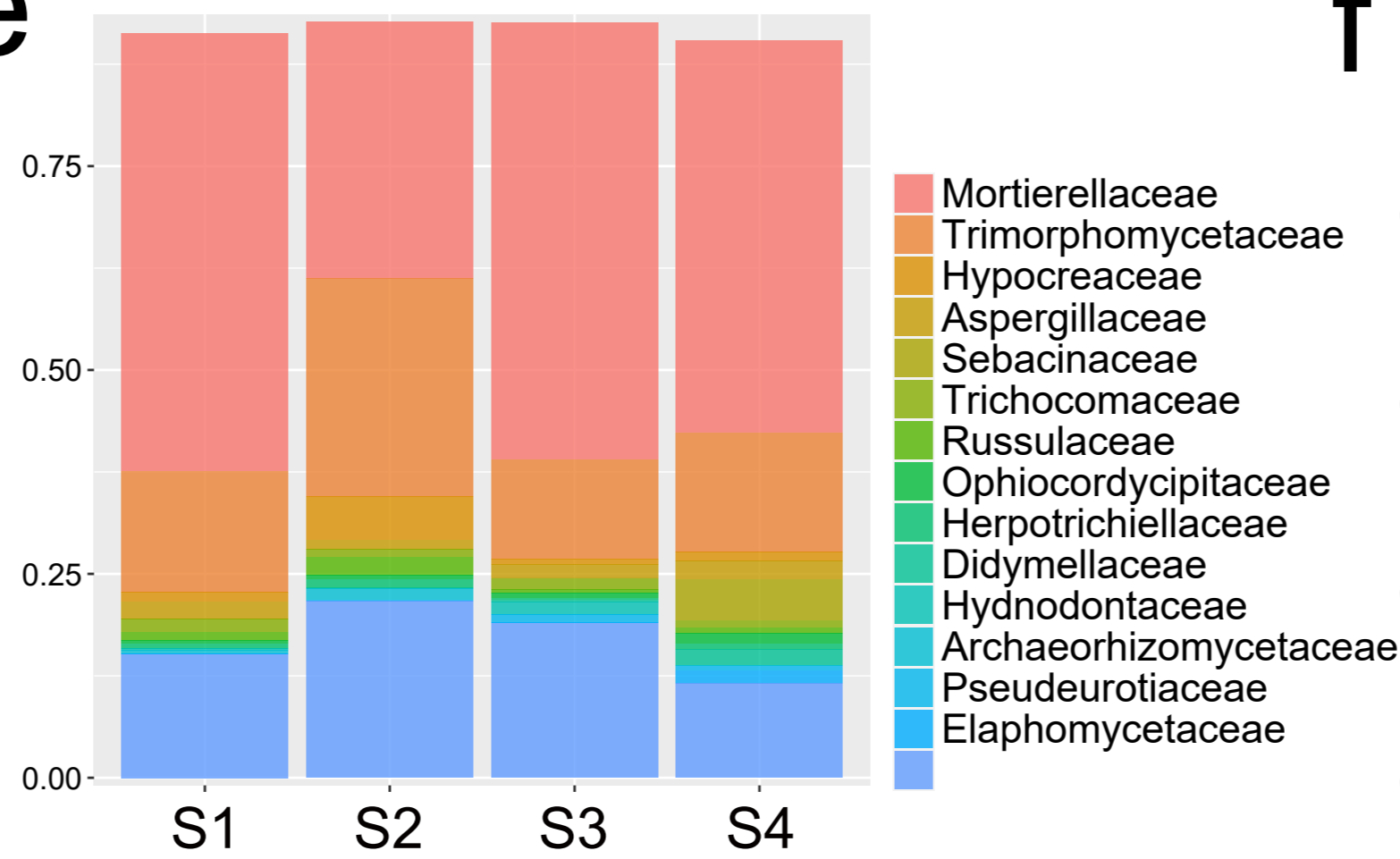

# f

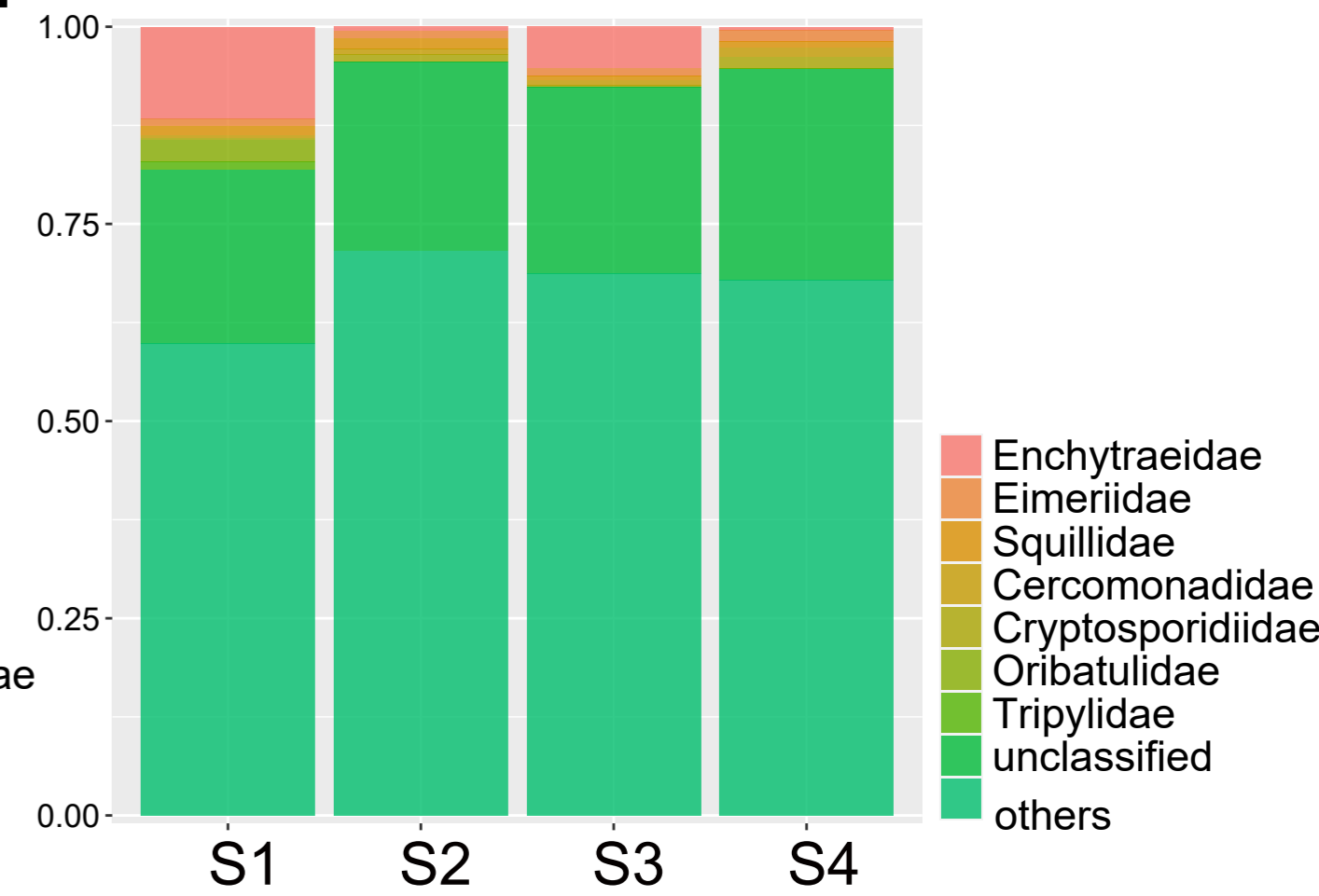

Supplement: Figure_S2_wraf191 [file figure_s2_wraf191.pdf]

a

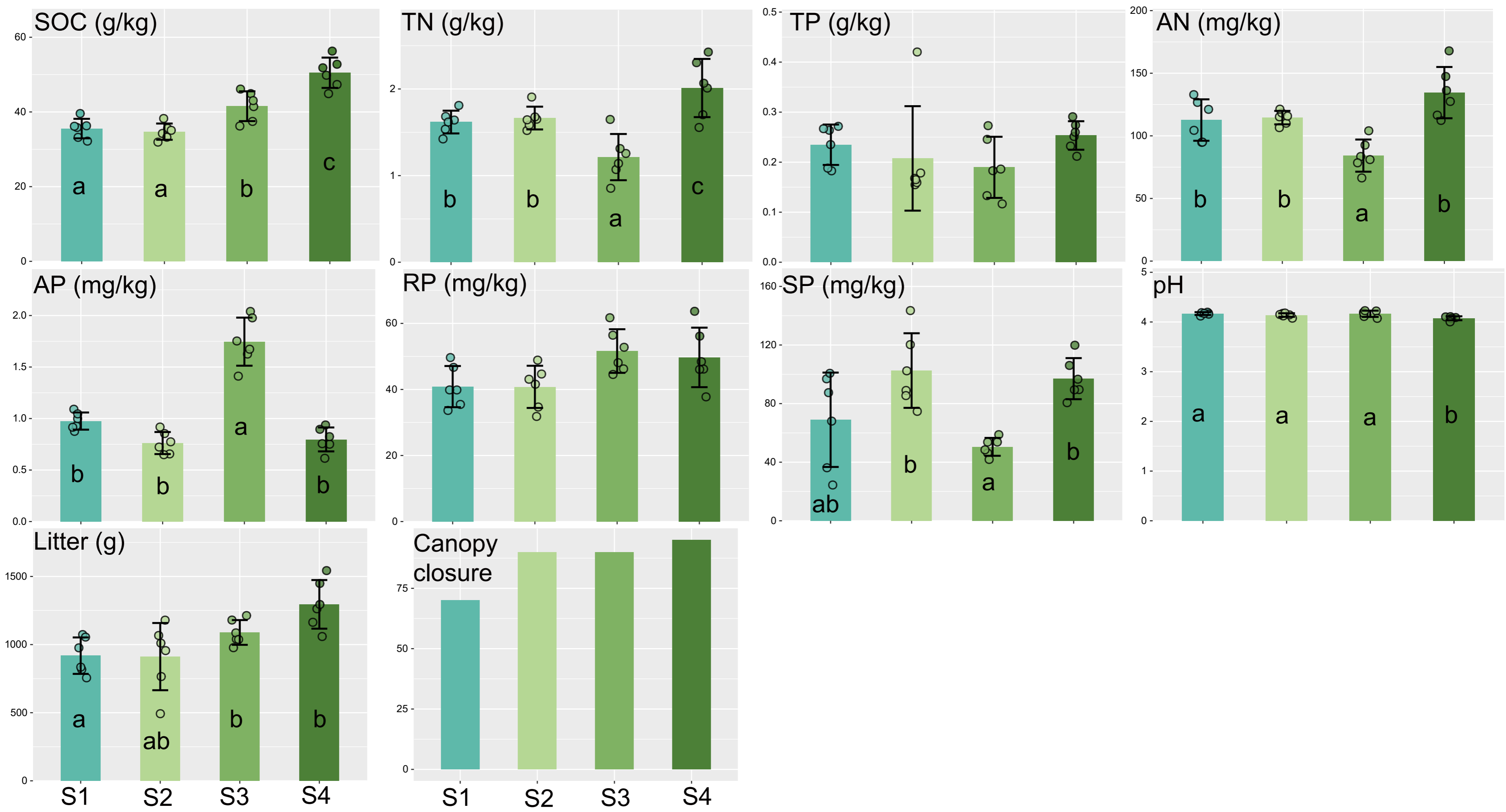

b

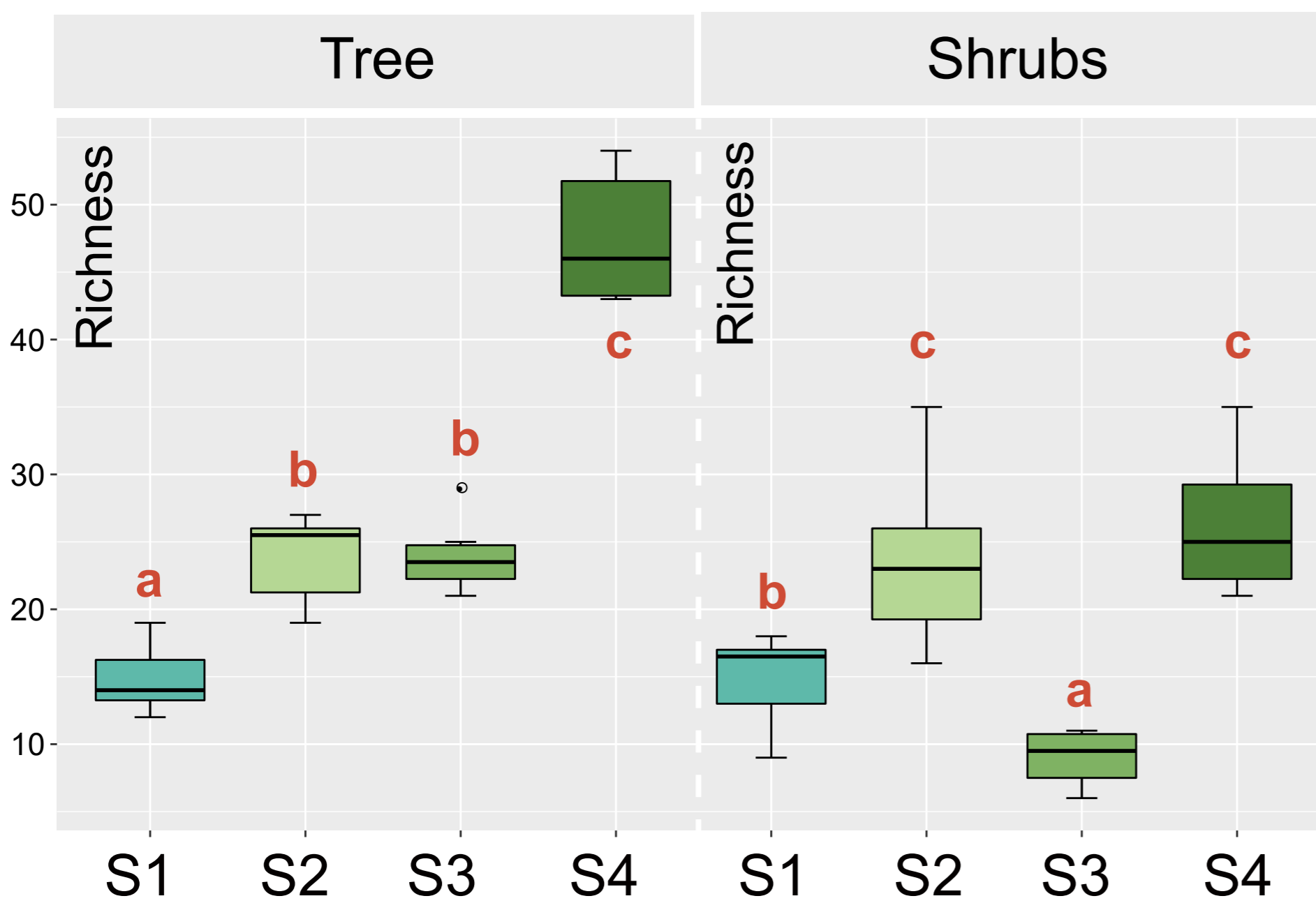

Supplement: Figure_S3_wraf191 [file figure_s3_wraf191.pdf]

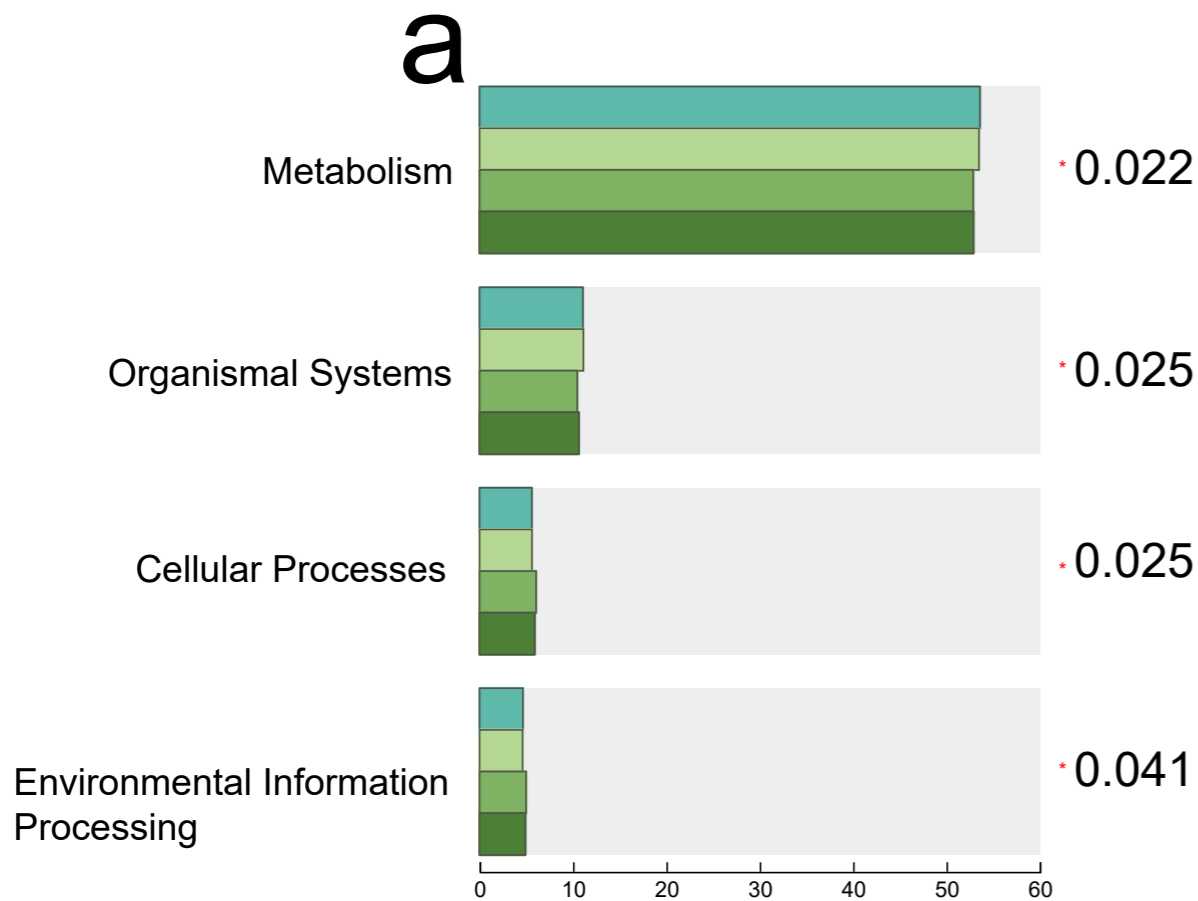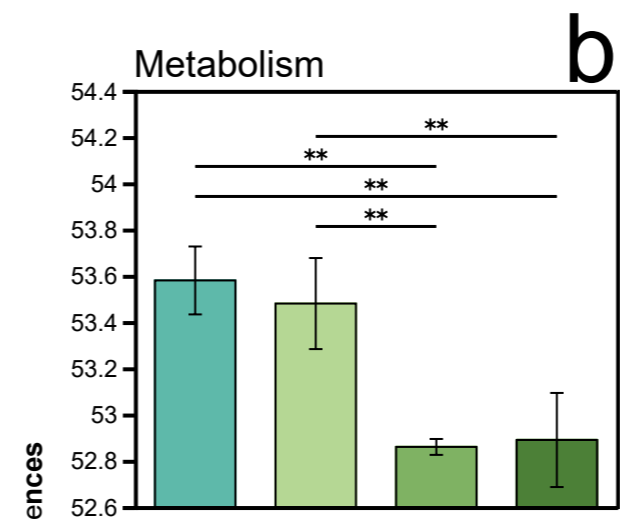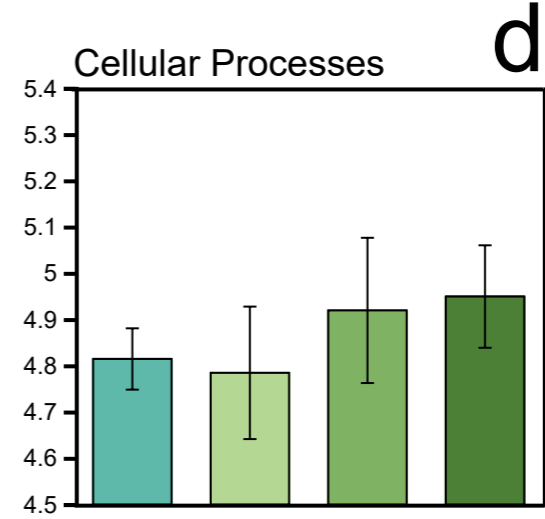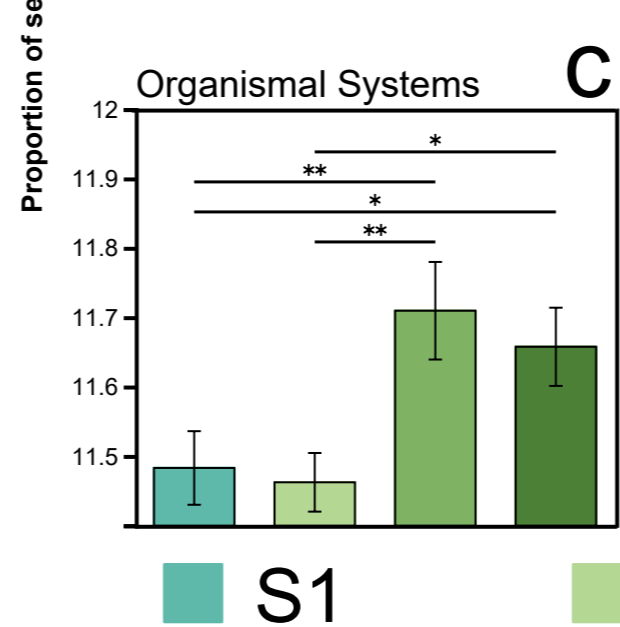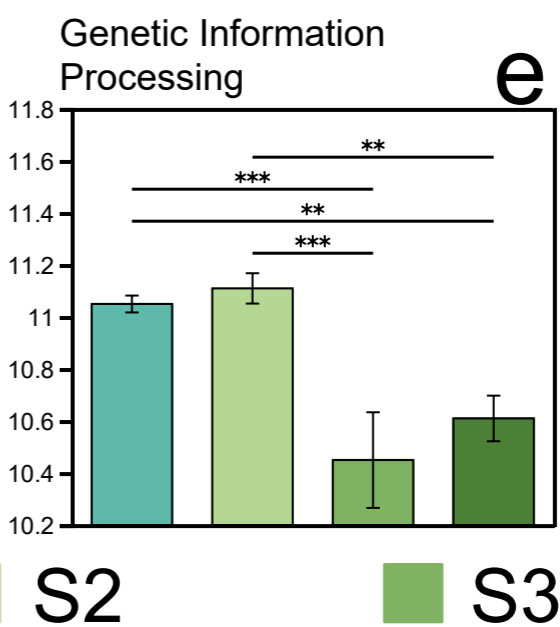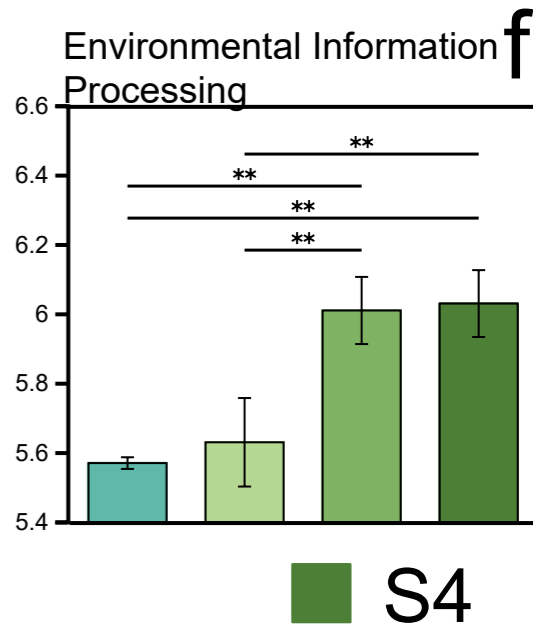

Supplement: Figure_S5_wraf191 [file figure_s5_wraf191.pdf]

**a**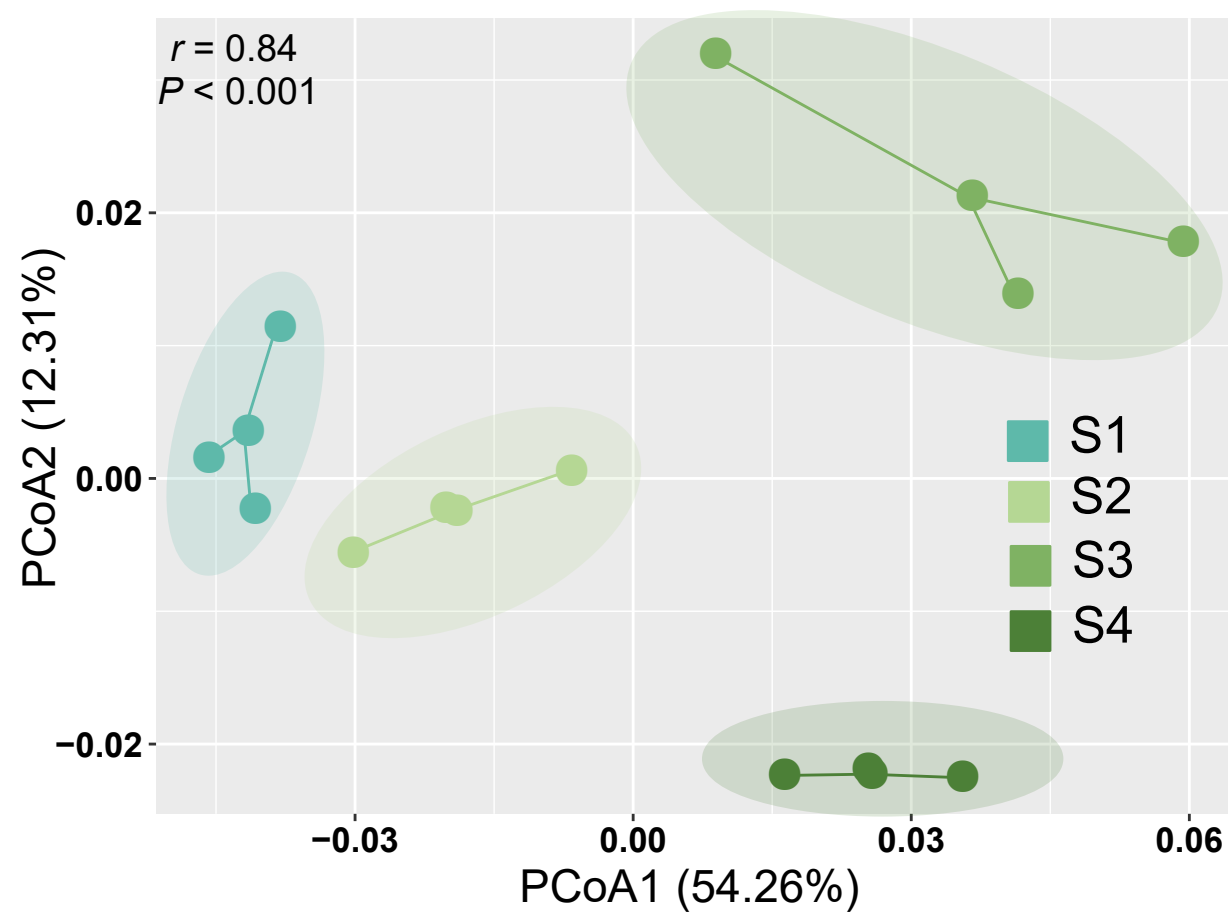**b**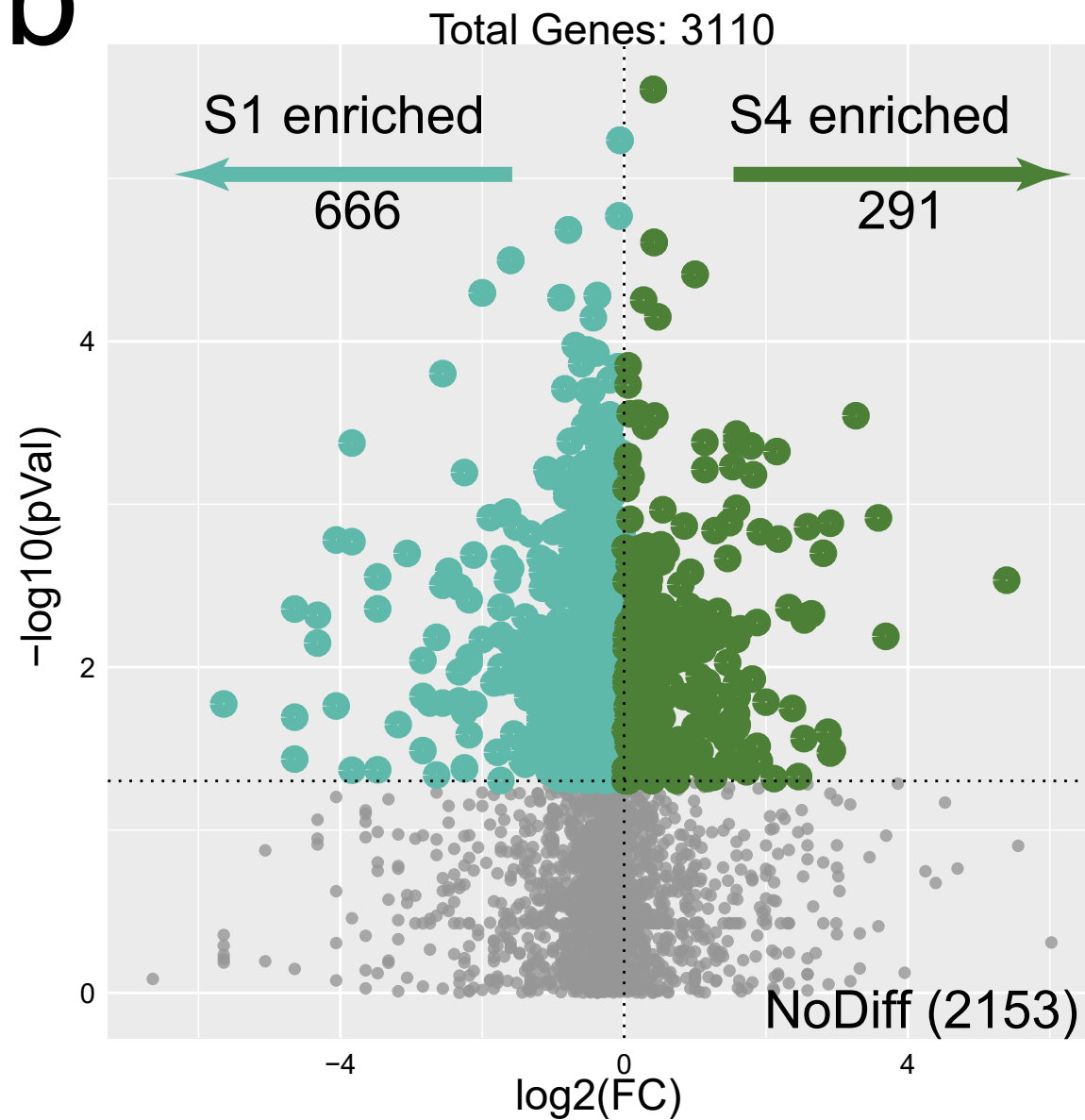

Supplement: Figure_S6_wraf191 [file figure_s6_wraf191.pdf]

# Carbon metabolism

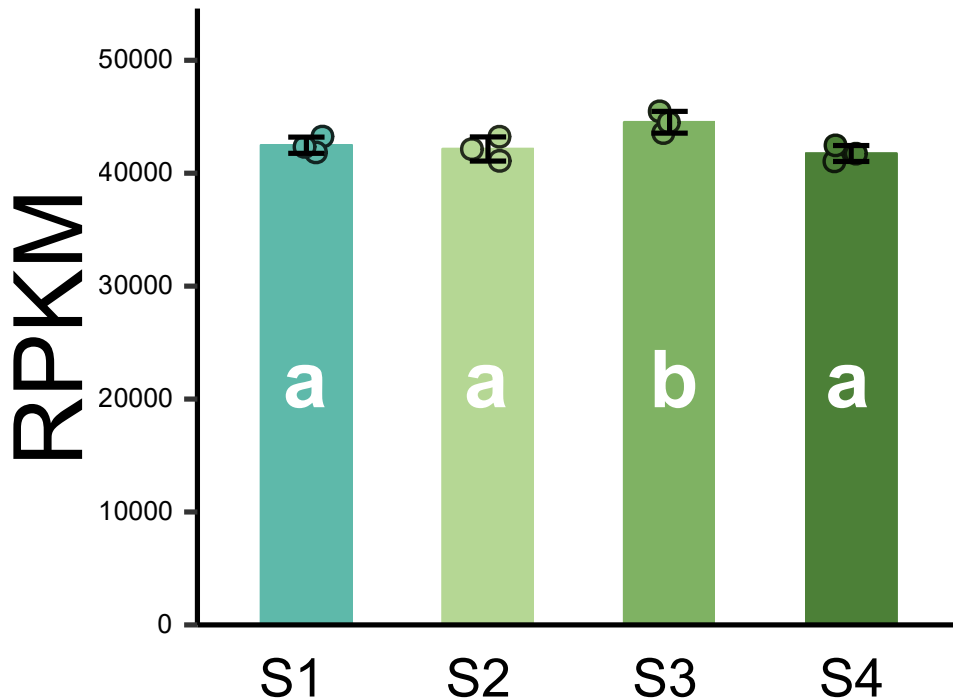

Supplement: Figure_S7_wraf191 [file figure_s7_wraf191.pdf]

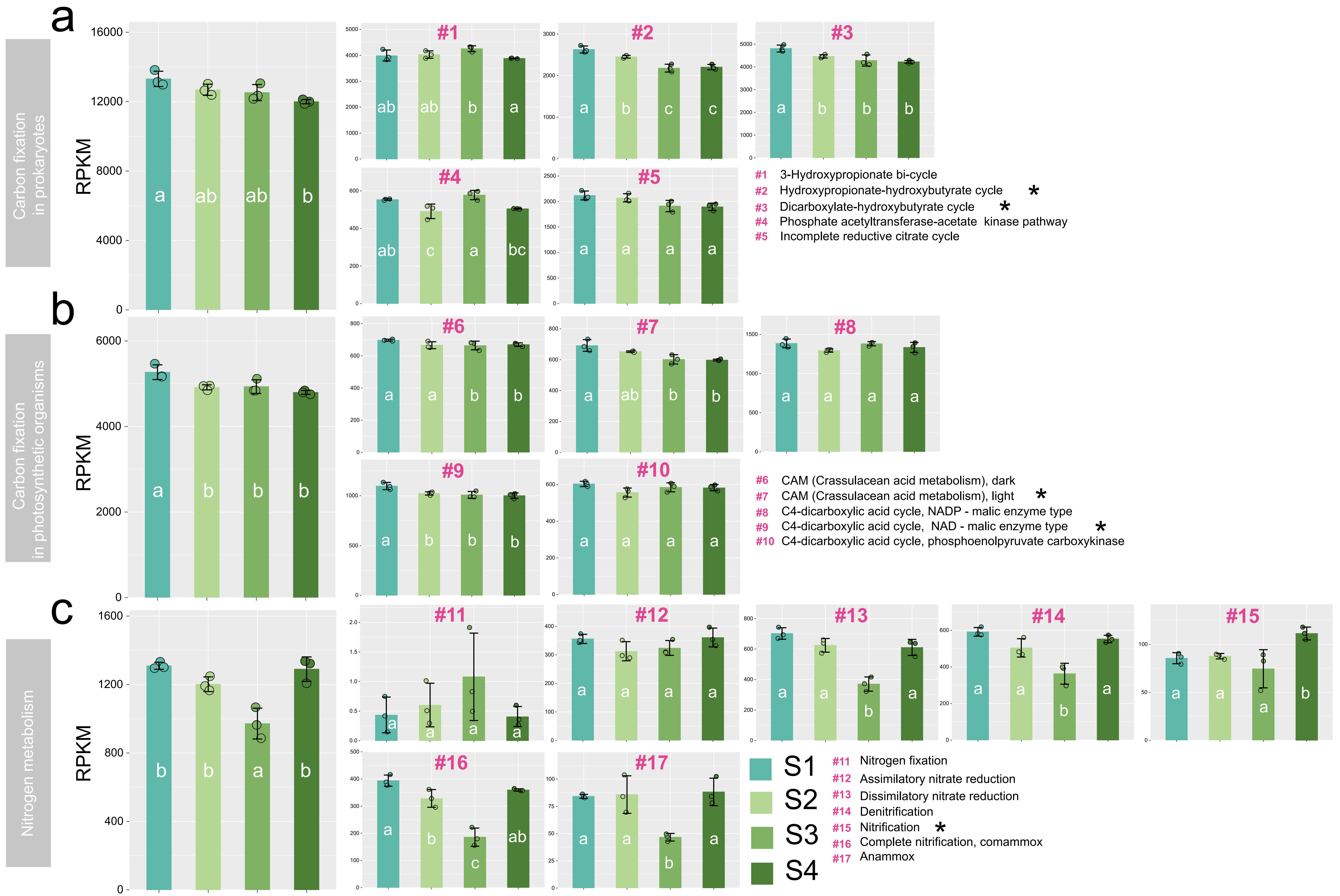

Supplement: Figure_S8_wraf191 [file figure_s8_wraf191.pdf]

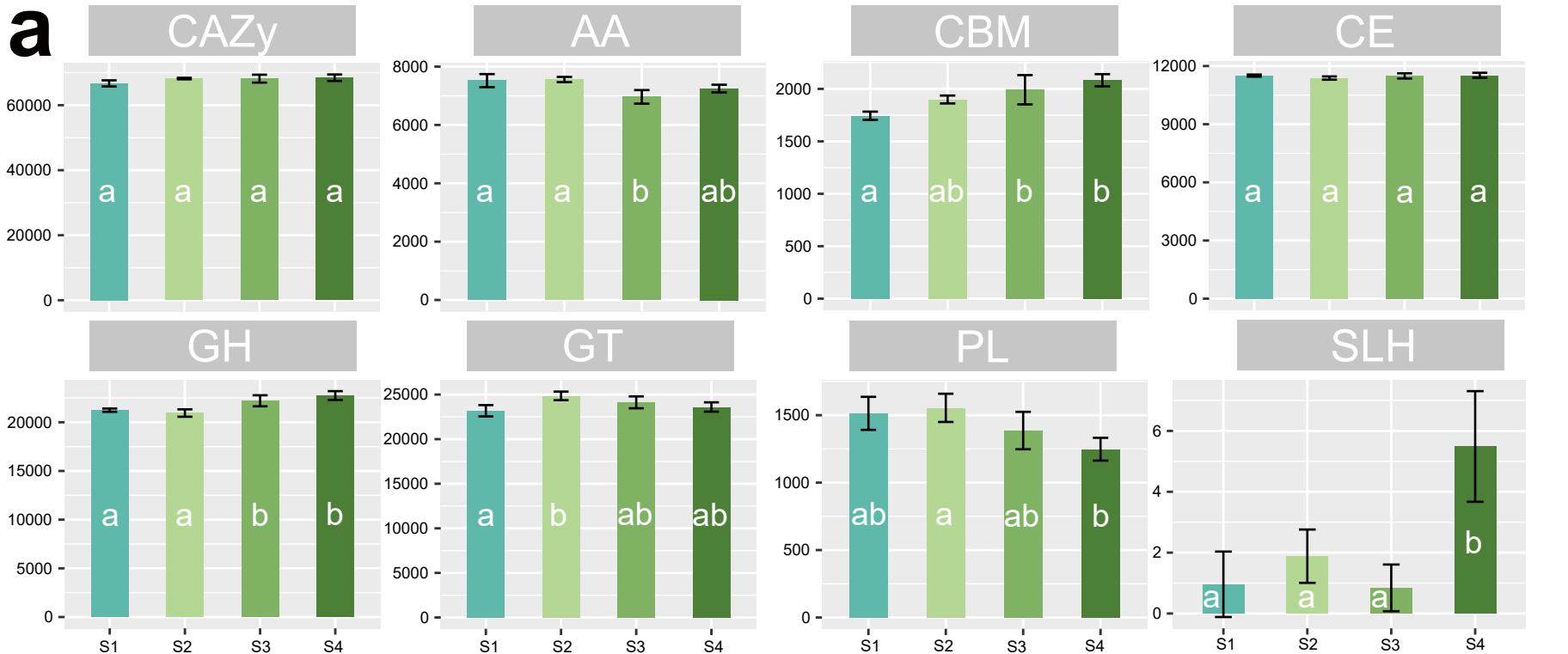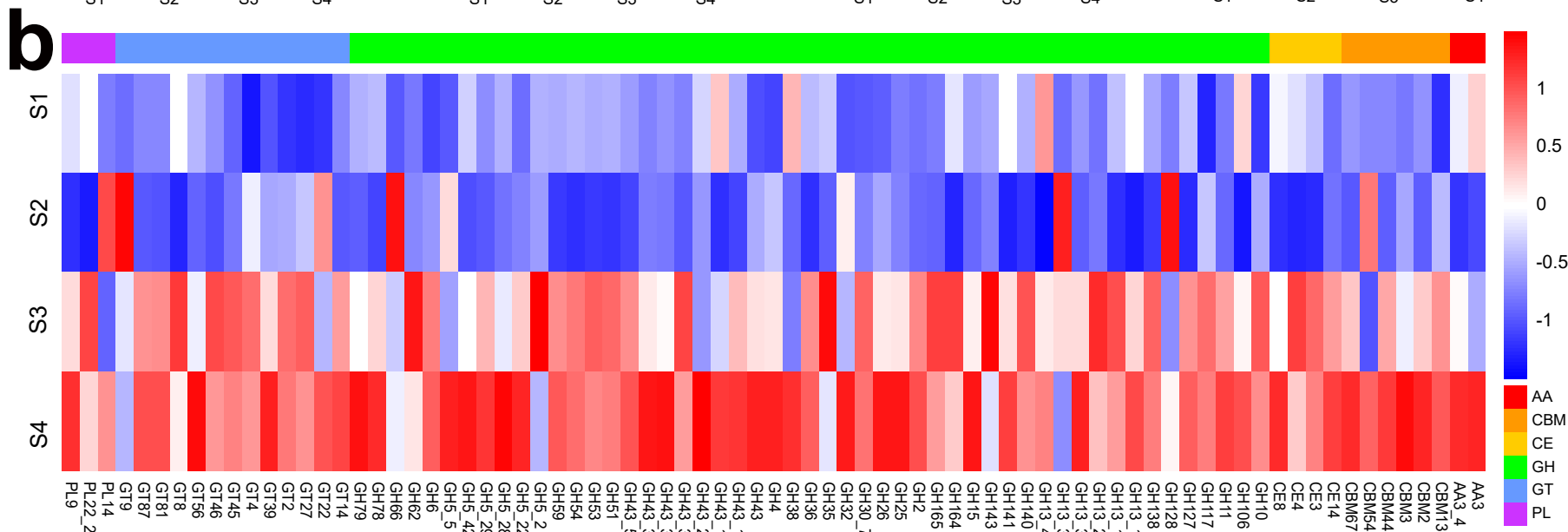

Supplement: Figure_S9_wraf191 [file figure_s9_wraf191.pdf]

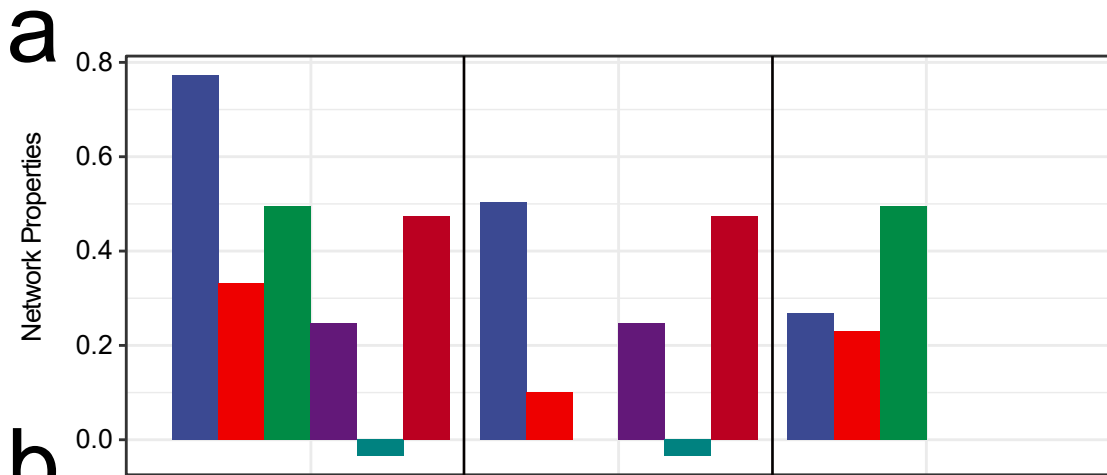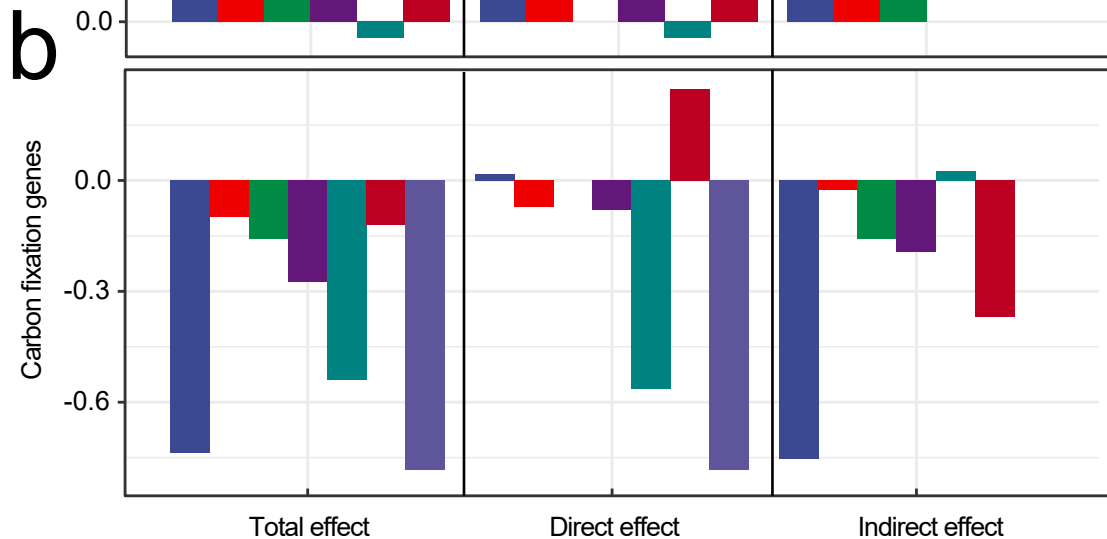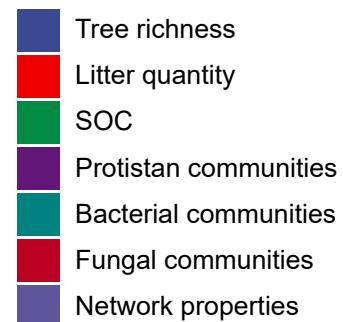

Supplement: Figure_S10_wraf191 [file figure_s10_wraf191.pdf]

a

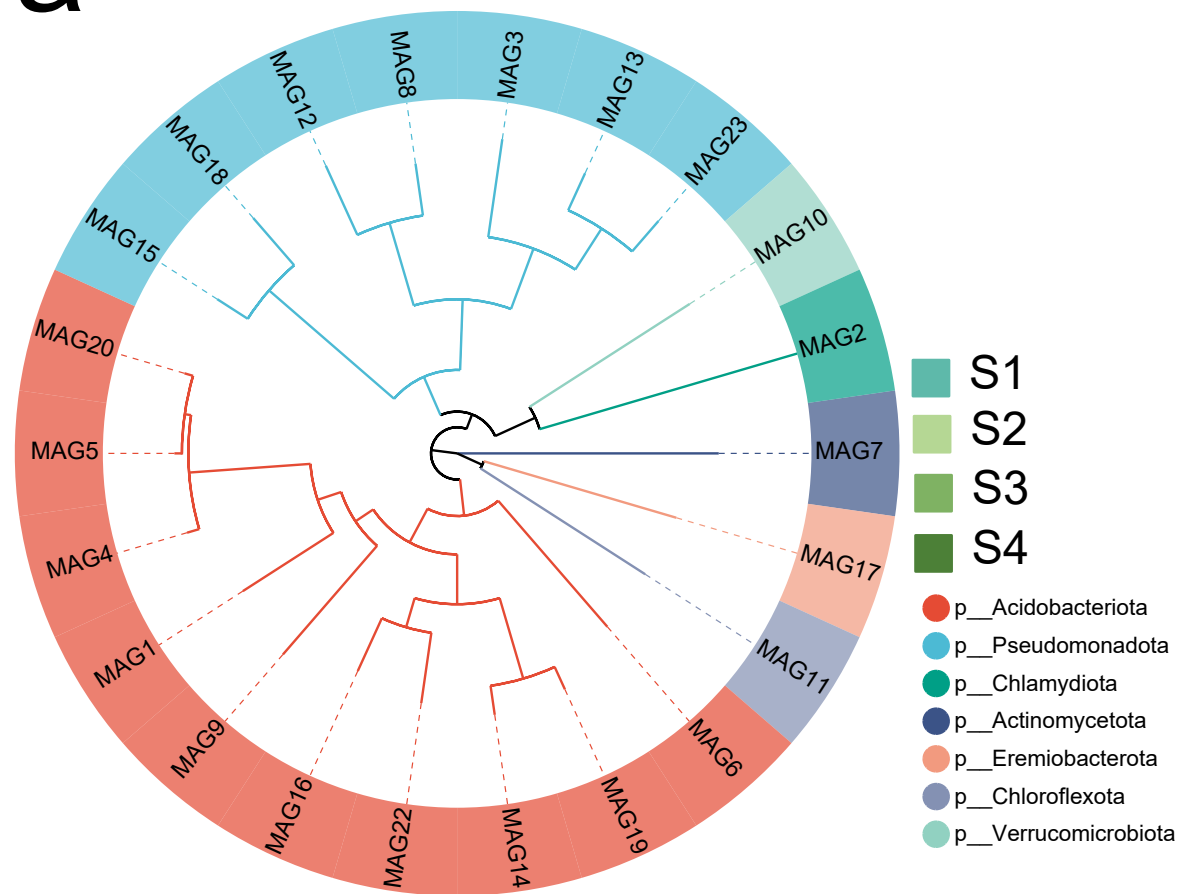

b

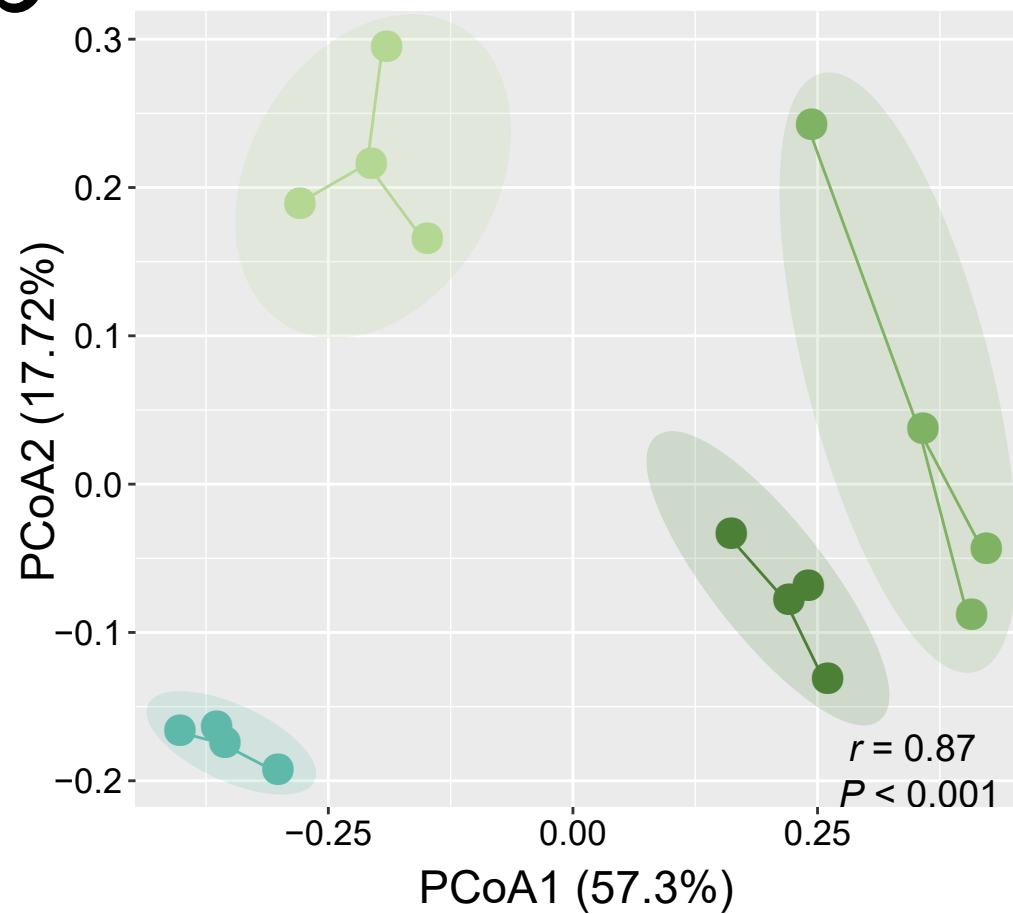

Supplement: Figure_S11_wraf191 [file figure_s11_wraf191.pdf]

a

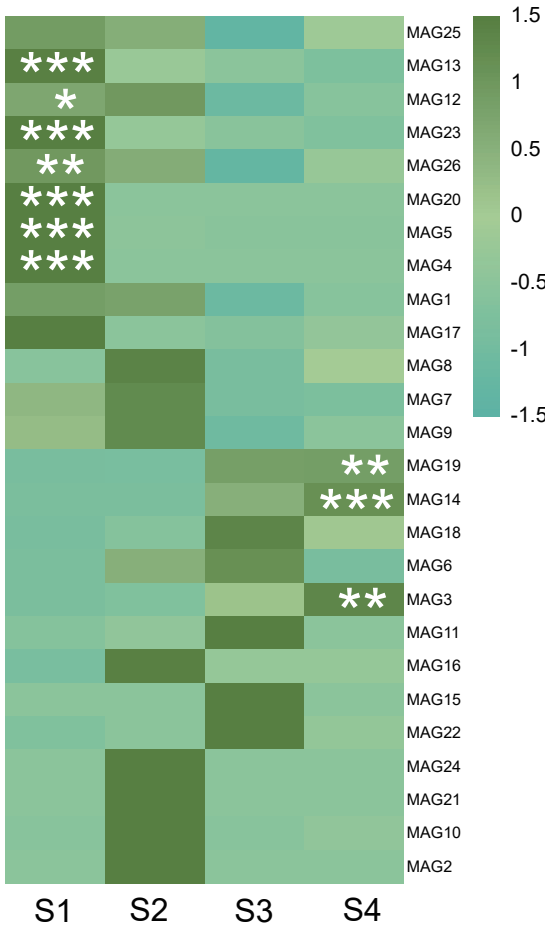

b

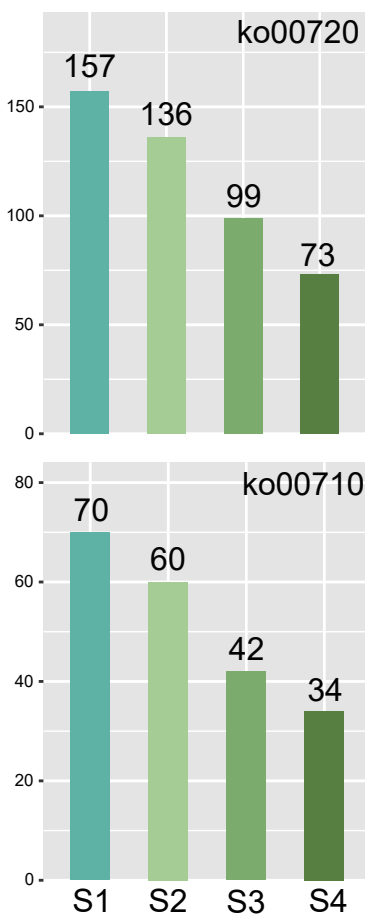

c

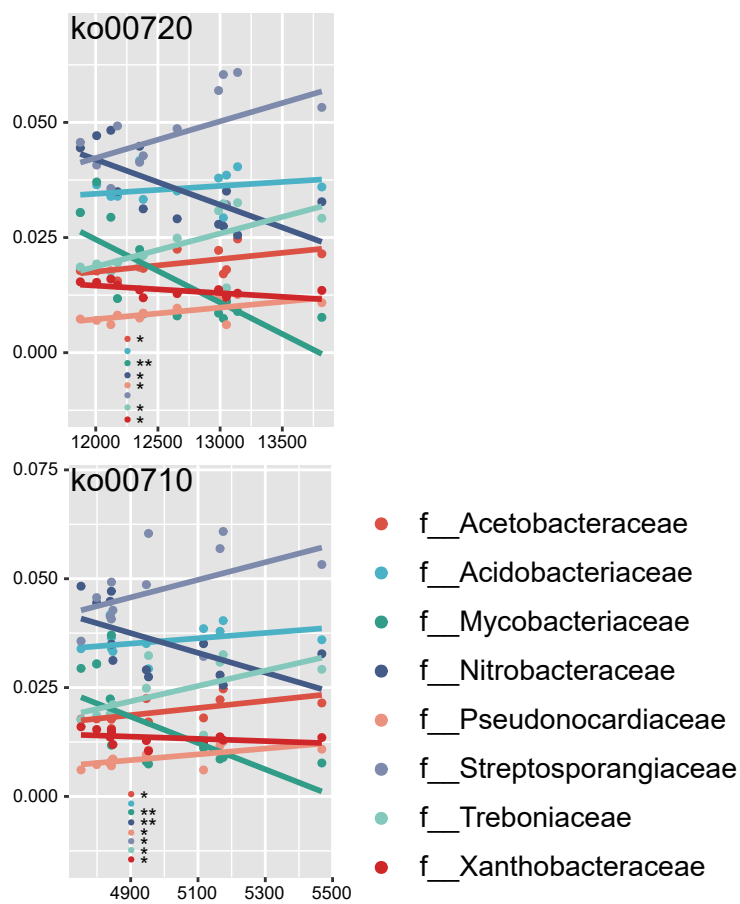

Supplement: Figure_S12_wraf191 [file figure_s12_wraf191.pdf]
