# Supplementary material for: Multi-scale evidence for declining microbial carbon fixation along forest succession gradients
Source: ISME J. 2025 Aug 24;19(1):wraf191. doi: 10.1093/ismejo/wraf191 (PMC12448444; doi:10.1093/ismejo/wraf191)
Supplement: Supplementary_materials_wraf191 [file supplementary_materials_wraf191.docx]

**Multi-Scale Evidence for Declining Microbial Carbon Fixation Along Forest Succession Gradients**

**Running title: Microbial CO_2_ Fixation Along Succession**

Shu-Yi-Dan Zhou ^a, b, c^, Zhiyang Lie ^a, b, c^, Chaotang Lei ^a, b, c^, Qi Zhang ^e^, Xujun Liu ^a, b, c^, Guopeng Wu ^a, b, c^, Roy Neilson ^f^, Fu-Yi Huang ^d^, Guowei Chu ^a, b, c^, Ze Meng ^a, b, c^, Dong Zhu ^d*^, David T Tissue ^g^, Josep Peñuelas ^h, i^, Juxiu Liu ^a, b, c*^

1. Guangdong Provincial Key Laboratory of Applied Botany, South China Botanical Garden, Chinese Academy of Sciences, 723Xingke Road, Tianhe District, Guangzhou 510650, China
2. National Ecological Science Data Center Guangdong Branch, 723Xingke Road, Tianhe District, Guangzhou 510650, China
3. Guangdong Province Data Center of Terrestrial and Marine Ecosystems Carbon Cycle，723Xingke Road, Tianhe District, Guangzhou 510650, China
4. State Key Laboratory of Regional and Urban Ecology, Ningbo Observation and Research Station, Institute of Urban Environment, Chinese Academy of Sciences, Xiamen 361021, China
5. Institute for Advanced Study, Shaoxing University, Shaoxing 312000, P. R. of China
6. Ecological Sciences, The James Hutton Institute, Dundee, DD2 5DA, Scotland, UK
7. Hawkesbury Institute for the Environment, Western Sydney University, Hawkesbury Campus, Richmond NSW 2753, Australia
8. CSIC, Global Ecology Unit CREAF- CSIC-UAB, Bellaterra, Barcelona, 08193, Catalonia, Spain
9. CREAF, Cerdanyola del Vallès, Barcelona 08193, Catalonia, Spain

* Corresponding author: Juxiu Liu, E-mail address: [ljxiu@scbg.ac.cn](mailto:ljxiu@scbg.ac.cn)

Dong Zhu, E-mail address: dzhu@iue.ac.cn

**Contents**

**Supplementary Notes:**

**Note S1.** Geospatial details of the sampling locations.

**Note S2.** High-throughput sequencing and analysis.

**Note S3.** Details of ¹³CO₂ Labelling for Soil CO₂ Fixation Measurement.

**Note S4.** Statistics and Visualization for Microbial Community.

**Supplementary Figures:**

**Figure S1.** Shannon diversity of bacterial, fungal and protistan communities, respectively.

**Figure S2.** Composition of bacterial, fungal and protistan communities at phylum (a) and family (b) level.

**Figure S3.** Profiles of soil organic matter, total nitrogen (TN), total phosphorus (TP), available nitrogen (AN), available phosphorus (AP), rapidly available potassium (RP), slowly available potassium (SP), pH, and litter quantity in different stages of forest succession. Tree and Shrubs richness in forest succession (b).

**Figure S4.** The ratio of positive to negative network correlations (a). The keystone species in different networks (b). Shared notes in different stages of succession (c).

**Figure S5.** The KEGG pathways, Metabolism (b), Organismal systems (c), Human Diseases (d), Genetic information processing (e), Environmental information processing (g), of forest soil under succession (* *P* ≤ 0.05，** *P* ≤ 0.01，*** *P* ≤ 0.001).

**Figure S6.** The Principal Coordinates Analysis (PCoA) analysis of KEGG functions (a), and a volcano plot illustrating the enrichment status of metabolism related functional genes (b).

**Figure S7.** The KEGG pathway of carbon metabolism under forest succession. Different letters indicate a significant difference at 0.05 level.

**Figure S8.** Functional potential of carbon and nitrogen cycle.

**Figure S9.** Annotated carbohydrate-active enzyme.

**Figure S10.** Cluster diagram of metagenome-assembled genomes (MAGs) at phylum level. The PCoA analysis of MAGs in different stages of succession.

**Figure S11.** Profiles of metagenome-assembled genomes in carbon fixation.

**Figure S12.** Direction of total, direct and indirect effects of tree richness, fungal communities, litters, protistan communities, bacterial communities and SOC on network stability and microbial carbon fixation genes.

**Supplementary Tables:**

**Table S1.** Comparison of microbe networks constructed from empirical data and 100 random assemblages under subtropical forest succession.

**Table S2.** Topological parameters of microbial networks under subtropical forest

**References**

**Supplementary Notes**

**Note S1. Geospatial details of the experimental site.**

The reserve has a typical subtropical monsoon climate with an average annual temperature of 22.3 ℃ and an average annual rainfall of 1900 mm. The soil type is latosolic red soil. Soil pH ranges from 4.0 to 4.9.

The study site included four forest successional stages: The Pine Forest (S1), Mixed Pine and Broadleaf Forest (S2), elder Mixed Pine and Broadleaf Forest (S3), and Monsoon Evergreen Broadleaf Forest (S4), approximately ~60, ~100, ~150, and >400 years old, that were located at elevations of 50–150 m, 300–350 m, 100–200 m, and 580–620 m, respectively. Among these, the pine forest was a plantation that was planted in the 1950s. In 1978, one-hectare permanent plots were established at random locations within each forest successional stage, with each plot typically separated by approximately 1.7 kilometers. Plots shared similar soil parent material and hydrothermal conditions. The understory vegetation for each forest successional stage has been reported previously ^1^.

**Note S2. High-throughput sequencing and analysis.**

High-throughput amplicon sequencing was employed to characterize microbial diversity. Amplicon sequencing to identify members of the soil microbial communities, following extraction of DNA by Majorbio (Shanghai, China) using a Fast DNA Spin Kit for soil (MP Biomedicals, USA) according to the manufacturer’s instructions. Several gene targets were characterized: the 16S hypervariable region V3 (ACTCCTACGGGAGGCAGCA)-V4 (GGACTACHVGGGTWTCTAAT) ^2^, ITS1 (CTTGGTCATTTAGAGGAAGTAA) -ITS2 (GCTGCGTTCTTCATCGATGC) ^3^, and 18s rRNA TAReukFWD1F (CCAGCASCYGCGGTAATTCC) - TAReukREV3R (ACTTTCGTTCTTGATYRA) ^4^ for forest soil bacterial, fungal and protistan communities. Reaction volumes, conditions, and procedures were as previously reported. Sequence analysis used Quantitative Insights Into Microbial Ecology 2 (QIIME 2) ^5^ in combination with the DADA2 pipeline for identification of microbial ASVs ^6^. Figaro software was used to determine the optimal truncation position to improve reproducibility and standardization of the microbiome analysis ^7^.USEARCH was used to identify and discard chimeric sequences ^8^. Taxonomic classification of bacteria, fungi and protozoa used the Silva v138 ^9^, UNITE 8.0 ^10^ and PR2 (v5.0.0) databases ^11^, respectively To improve the reliability of downstream analyses, chloroplast was filtered out. We applied a taxonomic confidence level of > 97% to identify amplicon sequence variants (ASVs). Microbial alpha diversity was assessed using the Shannon diversity index, calculated with the ‘vegan’ package in R.

**Note S3. Details of ¹³CO₂ Labelling for Soil CO₂ Fixation Measurement.**

We estimated soil CO₂ fixation potential using the ¹³CO₂ labelling method 52. We used surface soil (0-10 cm) for the experiment, which is directly exposed to sunlight. Briefly, two 25 g fresh soil samples (sieved to 2 mm) were placed into 250 mL sealed incubation bottles and pre-incubated for 7 days at 60% water-holding capacity (WHC) at 25°C to restore microbial activity 53. One sample was freeze dried to be used as a control; the other labelled with ¹³CO₂. The incubation bottle was flushed with standard air for 5 min to replace the internal atmosphere, followed by injection of 12.5 mL of 99 atom% ¹³CO₂. The sample was then incubated at 25°C under a 12-hour light/12-hour dark cycle to simulate natural conditions, as the plot is situated in the South Asian subtropical region, where daylight and darkness each last approximately 12 hours. Air flushing and ¹³CO₂ replenishment were repeated every 5 days, with a total incubation period of 15 days. After incubation, the labelled soil sample was freeze dried and stored. Both soil samples were ground using a ball mill, and ¹³C content analyzed using an elemental analyzer coupled with isotope ratio mass spectrometry (EA-IRMS). The stable isotope abundance (¹³C atom%) was calculated relative to the international standard Pee Dee Belemnite (PDB). Soil ¹³CO₂ fixation potential was quantified by the increase in ¹³C atom% after incubation.

**Note S4. Statistics and Visualization for Microbial Community.**

To estimate the potential sources of members of the microbial communities across forest successional stages, we employed the FEAST (Fast Expectation Maximization for microbial Source Tracking) algorithm ^12^. FEAST is a statistical tool that uses microbial community data to infer the proportional contributions of defined 'source' communities to a given 'sink' community. In our analysis, early successional soils were treated as potential sources, and microbial communities from mid- and late-successional stages were considered sinks. The analysis was conducted using default parameters. The normalized stochasticity ratio (NST) of microbial communities was calculated using the "NST" R package with 1000 random computations, where NST values of > 50% indicates a community assembly dominated by stochastic processes, whereas NST values < 50% indicates deterministic dominance ^13^.

Microbial networks, with a series of associated network topology metrics, were constructed using the Molecular Ecological Network Analysis Pipeline (MENA; http://ieg4.rccc.ou.edu/mena/), with default settings. For each network node, within-module connectivity (Zi) and among-module connectivity (Pi) were calculated to determine its topological role. Based on established thresholds from previous studies, nodes were classified as module hubs (Zi ≥ 2.5, Pi < 0.62), connectors (Zi < 2.5, Pi ≥ 0.62), and network hubs (Zi ≥ 2.5, Pi ≥ 0.62), all of which were considered keystone nodes due to their potential ecological significance. Remaining nodes with low connectivity (Zi < 2.5, Pi < 0.62) were categorized as peripherals. Connectors are nodes that act as bridges between different modules in the network. They connect multiple modules, thus playing a crucial role in inter-module communication. Through these nodes, information, resources, or energy can flow between different modules, facilitating the overall coordination of the network. Connectors are not typically central nodes within any single module, but they are essential for maintaining communication between modules. Module hubs are the most important nodes within a specific module, typically having high connectivity (degree). They are primarily responsible for interactions between microbes within the same module. Module hubs play a "key role" within their module, enhancing the stability and functionality of the module by promoting cooperation or resource sharing among microbes. They are the core of each module. Network hubs are the most significant nodes in the entire network, usually connecting a large number of other nodes and playing a critical role between multiple modules. These nodes typically have a very high degree of connectivity and are central to the flow of information and resources across the network. Network hubs exert a dominant influence on the stability and function of the network. They may be the "core drivers" of the entire network. The Zi and Pi values were computed using MENA. To demonstrate that the constructed networks were non-random and representative, they were compared with 100 randomly assembled networks; with the confirmed non-random, constructed microbial networks visualized using Gephi (version 0.9.2). The "randomForest" package in R created random forest models based on microbial ASV level to predict different successional stages ^14^. Model predictive accuracy was assessed and cross-validated using the "rfcv" function.

Structural equation model (SEMs) was developed using SPSS AMOS Graphics (version 21) to identify the contributions of tree richness, soil organic carbon (SOC), litters, fungal, bacterial, and protozoan community structure to soil microbial network stability (SMNS), which ultimately influences carbon fixation potential. Data were standardized (Z-score) in SPSS prior to importing into AMOS Graphics software for modeling. Model validity was determined at p > 0.5 (Probability level), a goodness of fit index (GFI) > 0.9, and a root mean square error of approximation (RMSEA) < 0.05.

**Table S1.** Comparison of microbe networks constructed from empirical data and 100 random assemblages under subtropical forest succession.

| Network Indexes | S1 | | S2 | | S3 | | S4 | |
| --- | --- | --- | --- | --- | --- | --- | --- | --- |
|  | Constructed | Randomly assembled | Constructed | Randomly assembled | Constructed | Randomly assembled | Constructed | Randomly assembled |
| Average clustering coefficient | 0.243 | 0.012 +/- 0.008 | 0.224 | 0.019 +/- 0.011 | 0.319 | 0.045 +/- 0.009 | 0.344 | 0.057 +/- 0.007 |
| Average path distance | 3.491 | 7.345 +/- 0.835 | 6.587 | 4.739 +/- 0.161 | 9.537 | 3.693 +/- 0.060 | 4.843 | 3.032 +/- 0.028 |
| Geodesic efficiency | 0.459 | 0.194 +/- 0.016 | 0.23 | 0.257 +/- 0.006 | 0.186 | 0.304 +/- 0.003 | 0.259 | 0.364 +/- 0.002 |
| Harmonic geodesic distance | 2.18 | 5.182 +/- 0.433 | 4.351 | 3.901 +/- 0.096 | 5.384 | 3.289 +/- 0.036 | 3.862 | 2.750 +/- 0.017 |
| Centralization of degree | 0.027 | 0.027 +/- 0.000 | 0.049 | 0.049 +/- 0.000 | 0.082 | 0.082 +/- 0.000 | 0.108 | 0.108 +/- 0.000 |
| Centralization of betweenness | 0.012 | 0.216 +/- 0.070 | 0.188 | 0.168 +/- 0.035 | 0.21 | 0.134 +/- 0.015 | 0.083 | 0.084 +/- 0.007 |
| Centralization of stress centrality | 0.017 | 0.280 +/- 0.099 | 0.487 | 0.312 +/- 0.079 | 2.911 | 0.405 +/- 0.045 | 1.125 | 0.368 +/- 0.030 |
| Centralization of eigenvector centrality | 0.457 | 0.362 +/- 0.056 | 0.391 | 0.285 +/- 0.038 | 0.345 | 0.249 +/- 0.019 | 0.261 | 0.191 +/- 0.014 |
| Density | 0.017 | 0.017 +/- 0.000 | 0.024 | 0.024 +/- 0.000 | 0.018 | 0.018 +/- 0.000 | 0.028 | 0.028 +/- 0.000 |
| Reciprocity | 1 | 1.000 +/- 0.000 | 1 | 1.000 +/- 0.000 | 1 | 1.000 +/- 0.000 | 1 | 1.000 +/- 0.000 |
| Transitivity | 0.408 | 0.012 +/- 0.013 | 0.279 | 0.027 +/- 0.013 | 0.261 | 0.044 +/- 0.007 | 0.332 | 0.063 +/- 0.005 |
| Connectedness | 0.075 | 0.601 +/- 0.089 | 0.553 | 0.890 +/- 0.050 | 0.591 | 0.976 +/- 0.020 | 0.938 | 0.995 +/- 0.009 |
| Efficiency | 0.848 | 0.984 +/- 0.003 | 0.971 | 0.983 +/- 0.001 | 0.977 | 0.986 +/- 0.000 | 0.975 | 0.976 +/- 0.000 |
| Hierarchy | 0 | 0.000 +/- 0.000 | 0 | 0.000 +/- 0.000 | 0 | 0.000 +/- 0.000 | 0 | 0.000 +/- 0.000 |
| Lubness | 1 | 1.000 +/- 0.000 | 1 | 1.000 +/- 0.000 | 1 | 1.000 +/- 0.000 | 1 | 1.000 +/- 0.000 |
| Modularity(fast_greedy) | 0.901 | 0.786 +/- 0.014 | 0.775 | 0.627 +/- 0.012 | 0.808 | 0.484 +/- 0.007 | 0.662 | 0.330 +/- 0.006 |

S1: pine forest; S2: mixed pine-broadleaf; S3: mixed pine-broadleaf with elder; S4: monsoon evergreen-broadleaf.

**Table S2.** Topological parameters of microbial networks under subtropical forest succession.

| **Network Indexes** | **S1** | **S2** | **S3** | **S4** |
| --- | --- | --- | --- | --- |
| Total nodes | 84 | 112 | 223 | 253 |
| Total links | 68 | 149 | 447 | 880 |
| R square of power-law | 0.906 | 0.797 | 0.798 | 0.725 |
| Average degree | 1.619 | 2.661 | 4.009 | 6.957 |
| Average clustering coefficient | 0.232 | 0.224 | 0.319 | 0.344 |
| Average path distance | 1.58 | 6.587 | 9.537 | 4.843 |
| Geodesic efficiency | 0.777 | 0.23 | 0.186 | 0.259 |
| Harmonic geodesic distance | 1.287 | 4.351 | 5.384 | 3.862 |
| Maximal degree | 4 | 8 | 22 | 34 |
| Nodes with max degree | ASV950; ASV67; ASV113 | ASV96; ASV29 | ASV790 | ASV746 |
| Centralization of degree | 0.029 | 0.049 | 0.082 | 0.108 |
| Maximal betweenness | 14 | 1311.276 | 5676.954 | 3079.101 |
| Nodes with max betweenness | ASV950 | ASV29 | ASV695 | ASV584 |
| Centralization of betweenness | 0.004 | 0.188 | 0.21 | 0.083 |
| Maximal stress centrality | 20 | 3304 | 79750 | 40586 |
| Nodes with max stress centrality | ASV950 | ASV29 | ASV695 | ASV3 |
| Centralization of stress centrality | 0.006 | 0.487 | 2.911 | 1.125 |
| Maximal eigenvector centrality | 0.51 | 0.43 | 0.365 | 0.284 |
| Nodes with max eigenvector centrality | ASV67; ASV113 | ASV96 | ASV281 | ASV70 |
| Centralization of eigenvector centrality | 0.49 | 0.391 | 0.345 | 0.261 |
| Density | 0.02 | 0.024 | 0.018 | 0.028 |
| Reciprocity | 1 | 1 | 1 | 1 |
| Transitivity | 0.542 | 0.279 | 0.261 | 0.332 |
| Connectedness | 0.032 | 0.553 | 0.591 | 0.938 |
| Efficiency | 0.524 | 0.971 | 0.977 | 0.975 |
| Hierarchy | 0 | 0 | 0 | 0 |
| Lubness | 1 | 1 | 1 | 1 |

S1: pine forest; S2: mixed pine-broadleaf; S3: mixed pine-broadleaf with elder; S4: monsoon evergreen-broadleaf.


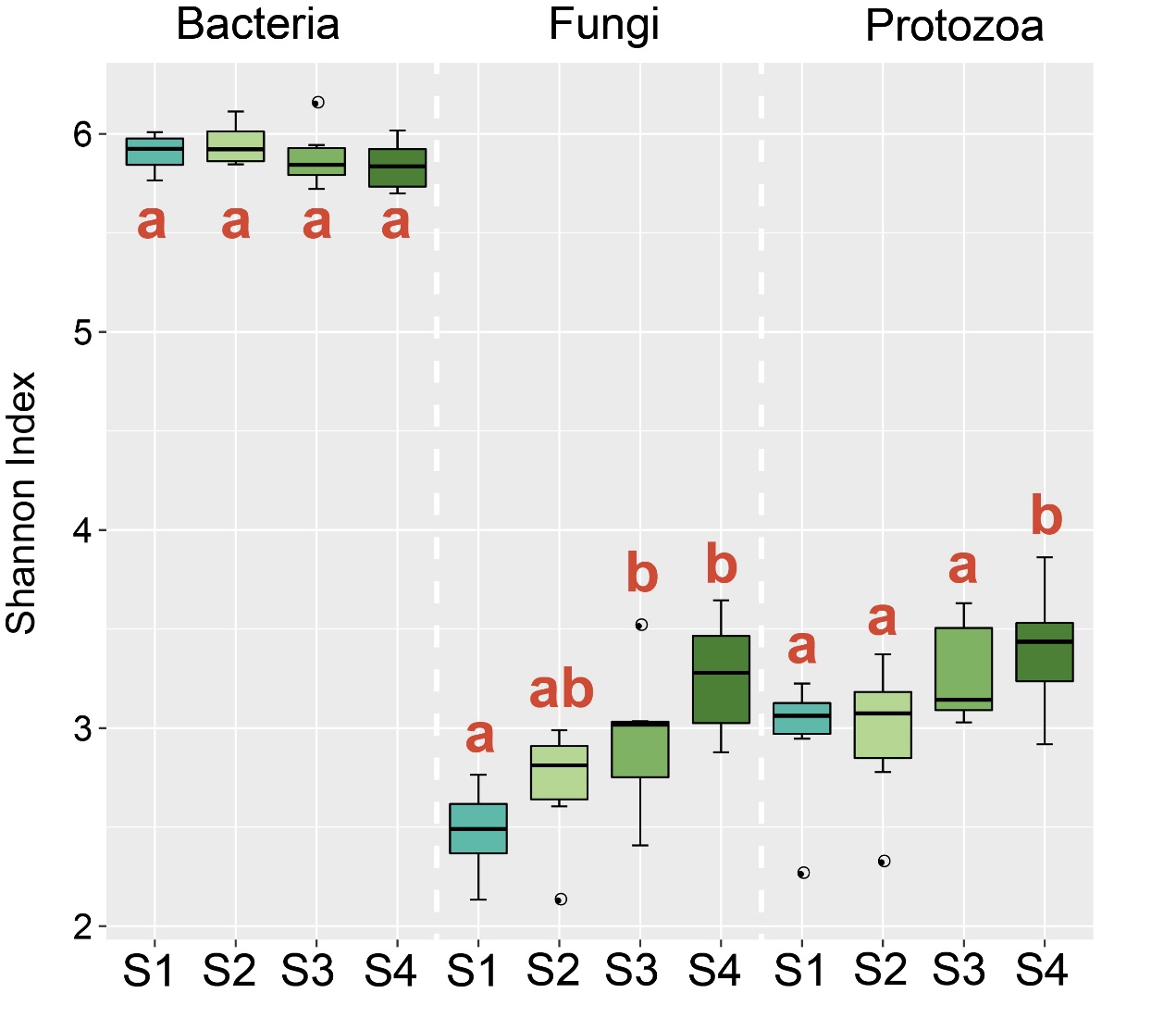


**Figure S1.** Shannon index of bacterial, fungal and protistan communities. Different letters indicate a significant difference at *P* < 0.05. Pine Forest (S1), Broadleaf Forest (S2), elder Mixed Pine and Broadleaf Forest (S3), and Monsoon Evergreen Broadleaf Forest (S4).


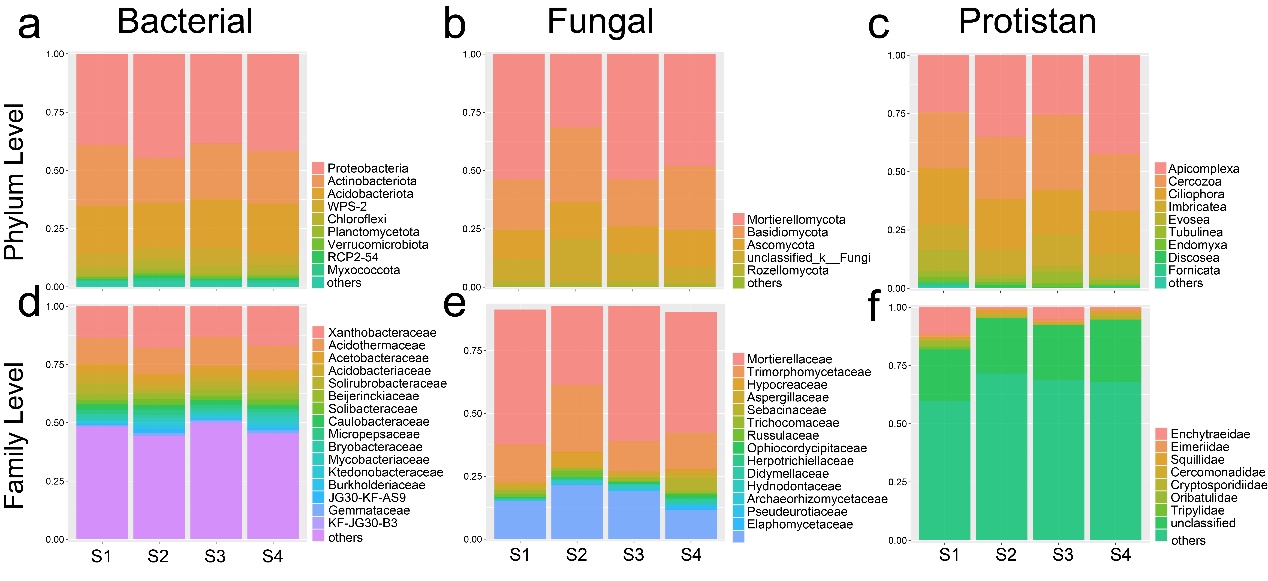


**Figure S2.** Composition of bacterial, fungal and protistan communities at phylum (a-c), and family (d-f) level for the different forest successional stages. Pine Forest (S1), Broadleaf Forest (S2), elder Mixed Pine and Broadleaf Forest (S3), and Monsoon Evergreen Broadleaf Forest (S4).


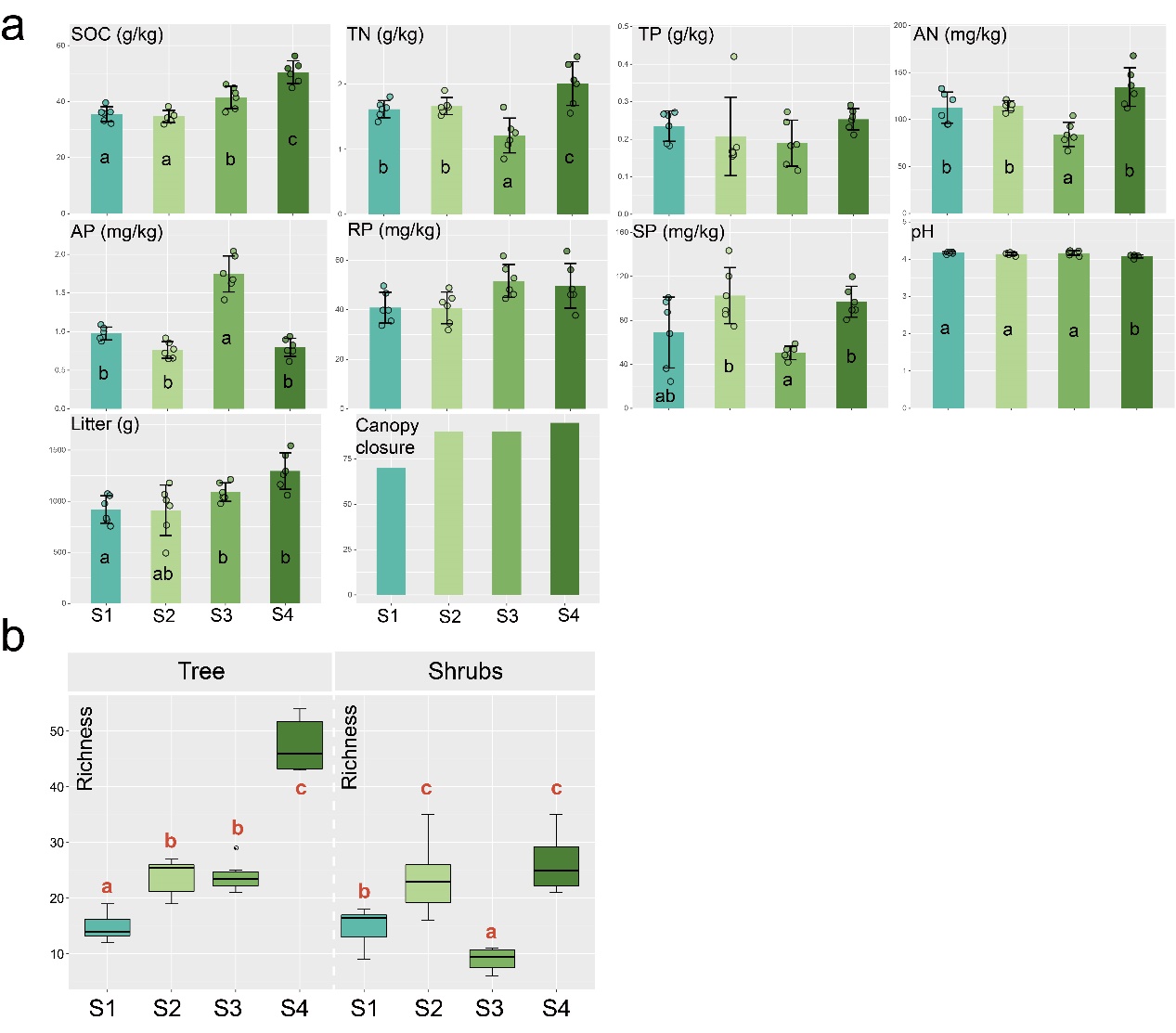


**Figure S3.** Soil organic carbon (SOC), total nitrogen (TN), total phosphorus (TP), available nitrogen (AN), available phosphorus (AP), rapidly available potassium (RP), slowly available potassium (SP), pH, litter quantity and canopy closure at different stages of forest succession. Different letters indicate a significant difference at *P* < 0.05. Pine Forest (S1), Broadleaf Forest (S2), elder Mixed Pine and Broadleaf Forest (S3), and Monsoon Evergreen Broadleaf Forest (S4) (a). Tree and shrub richness at different successional forest stages (b)


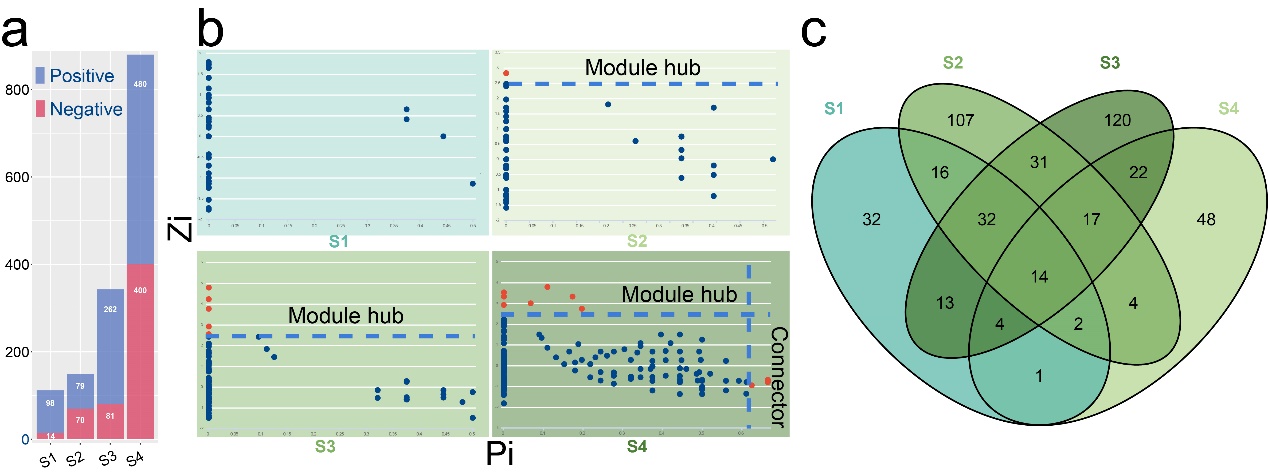


**Figure S4.** The ratio of positive to negative network correlations (a). Keystone species in different networks (b). Unique and shared nodes at different stages of forest succession (c). Pine Forest (S1), Broadleaf Forest (S2), elder Mixed Pine and Broadleaf Forest (S3), and Monsoon Evergreen Broadleaf Forest (S4).

**
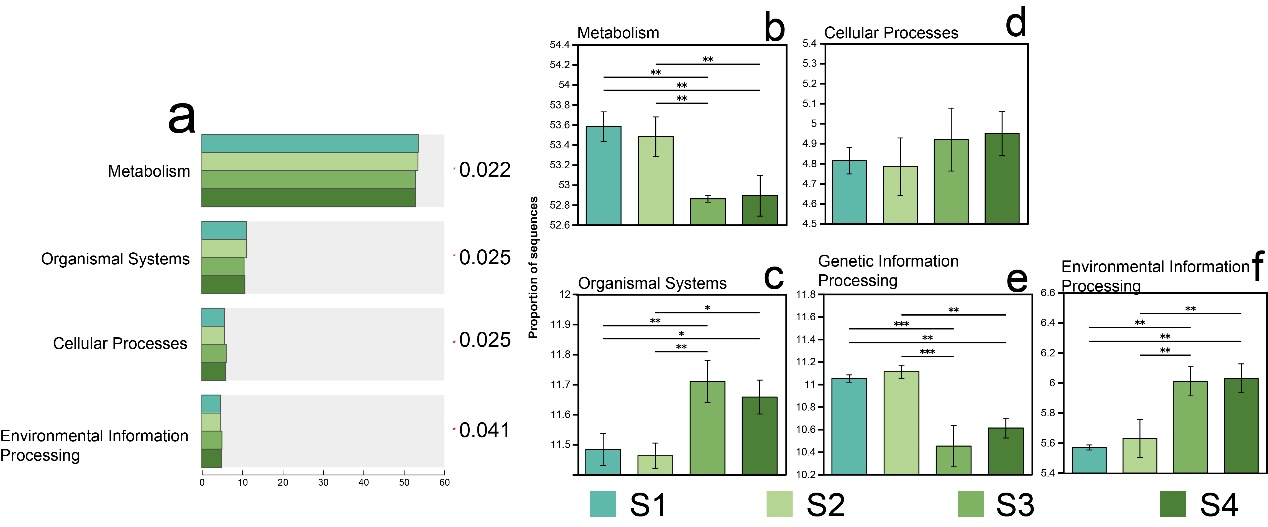
**

**Figure S5.** Profiles of overall pathways (a), KEGG pathways of Metabolism (b), Organismal systems (c), Cellular processes (d), Genetic information processing (e), Environmental information processing (f), under different forest successional stages (* *P* ≤ 0.05，** *P* ≤ 0.01，*** *P* ≤ 0.001). Pine Forest (S1), Broadleaf Forest (S2), elder Mixed Pine and Broadleaf Forest (S3), and Monsoon Evergreen Broadleaf Forest (S4).


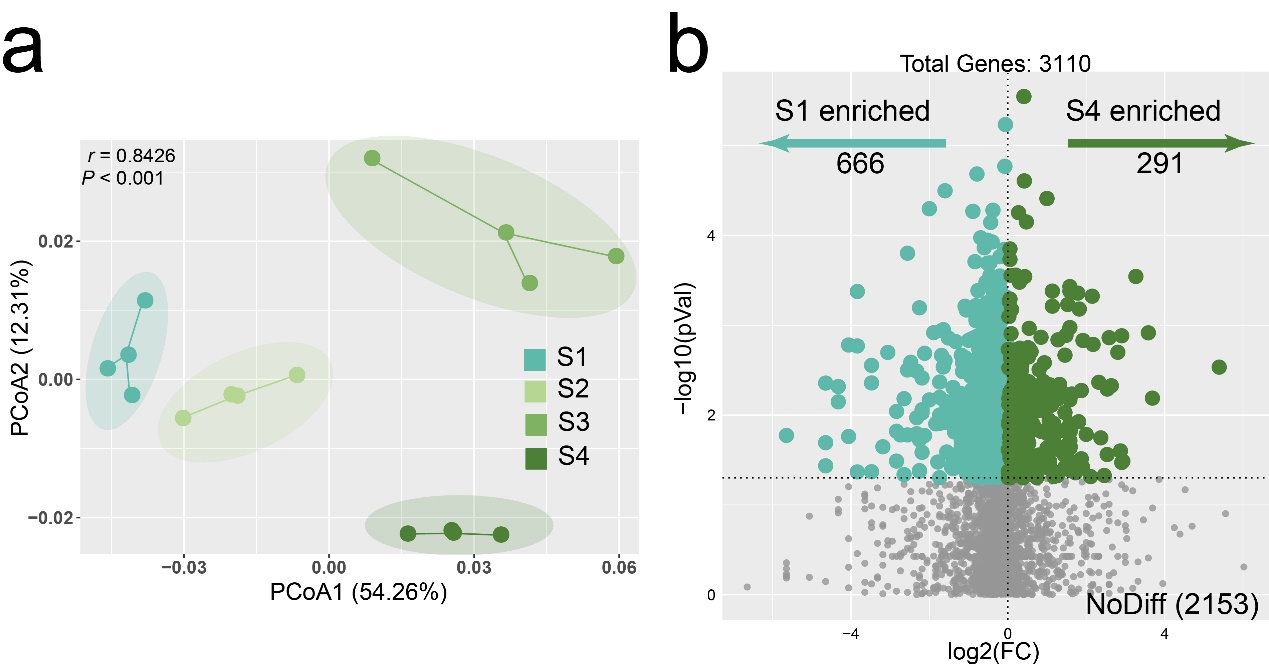


**Figure S6.** Principal Coordinates Analysis (PCoA) analysis of KEGG functions (a), and volcano plot illustrating the enrichment status of functional genes related to metabolism (b). Pine Forest (S1), Broadleaf Forest (S2), elder Mixed Pine and Broadleaf Forest (S3), and Monsoon Evergreen Broadleaf Forest (S4).


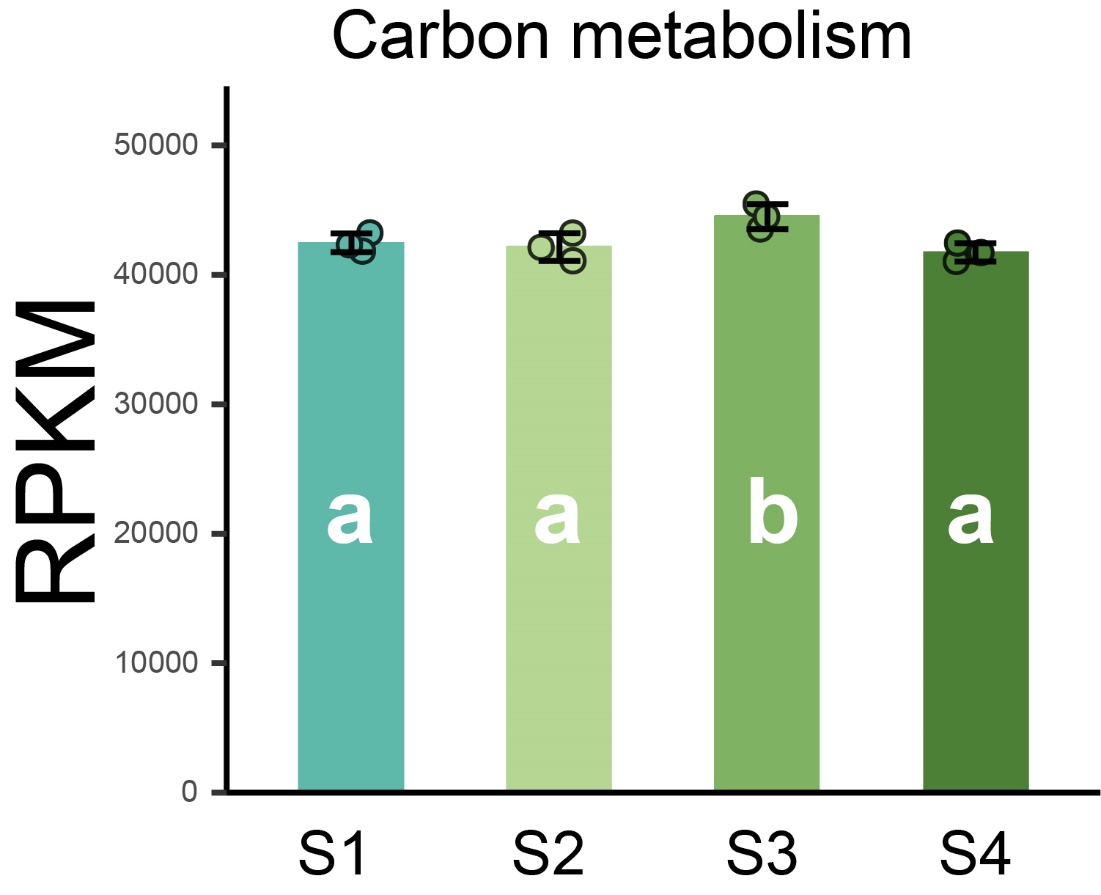


**Figure S7.** KEGG pathway of carbon metabolism under forest succession. Different letters indicate a significant difference at 0.05 level. Pine Forest (S1), Broadleaf Forest (S2), elder Mixed Pine and Broadleaf Forest (S3), and Monsoon Evergreen Broadleaf Forest (S4).

**
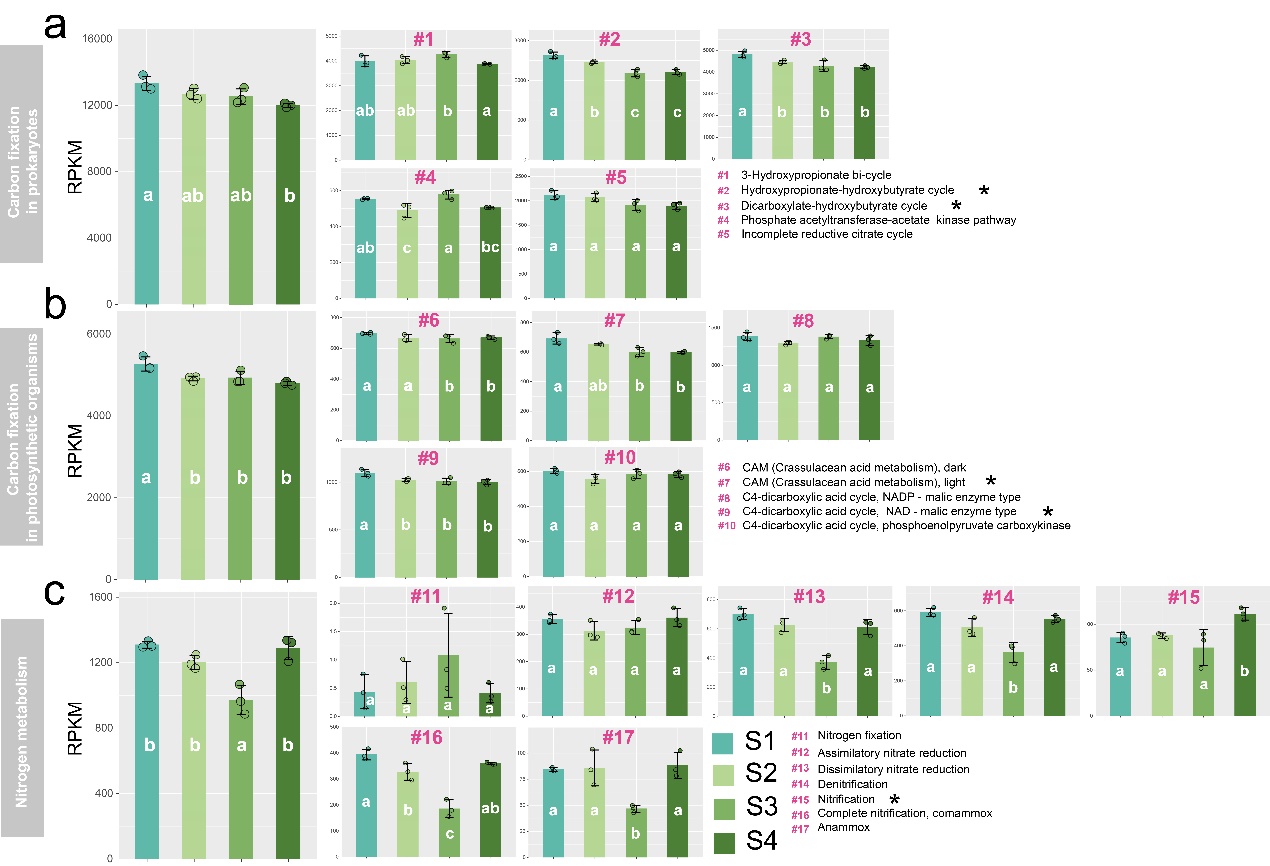
**

**Figure S8.** Functional potential and microbial carbon fixation in prokaryotes (a), carbon fixation in photosynthetic organisms (b), and nitrogen metabolism (c) across pathways and processes. * *P* ≤ 0.05, ** *P* ≤ 0.01, *** *P* ≤ 0.001. S1: pine forest; S2: mixed pine-broadleaf; S3: mixed pine-broadleaf with elder; S4: monsoon evergreen-broadleaf.


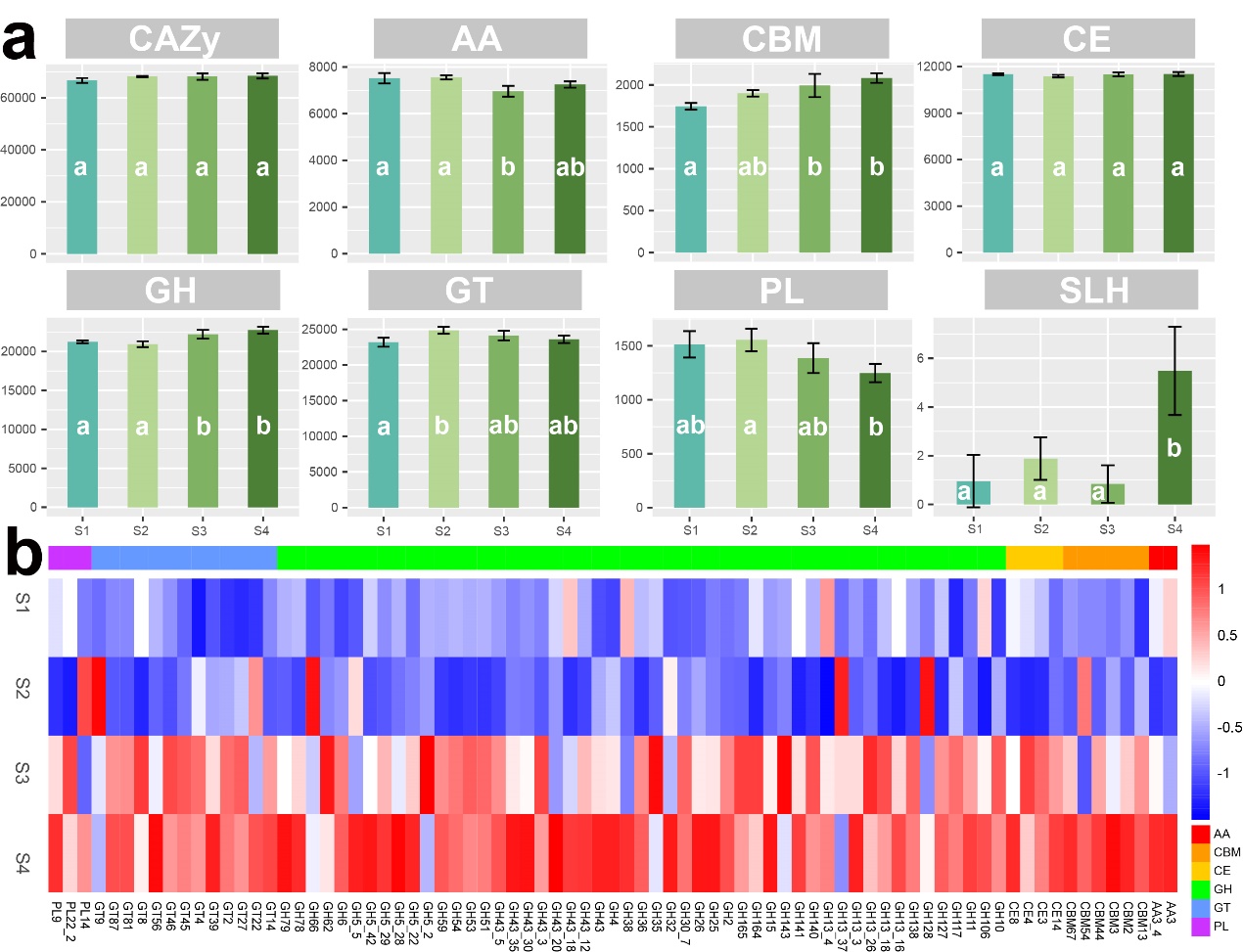


**Figure S9.** The abundance (RPKM) of CAZy classes (a). Heatmap depicting the successional-associated CAZy enzyme families, with abundance data normalized using z-scores (b). The CAZy classes including Auxiliary Activities (AA), Carbohydrate- Binding Modules (CBM), Carbohydrate Esterases (CE), Glycoside Hydrolases (GH), Glycosyl Transferases (GT), Polysaccharide Lyases (PL) and Cellulosome Modules (SLH). S1: pine forest; S2: mixed pine-broadleaf; S3: mixed pine-broadleaf with elder; S4: monsoon evergreen-broadleaf.

**
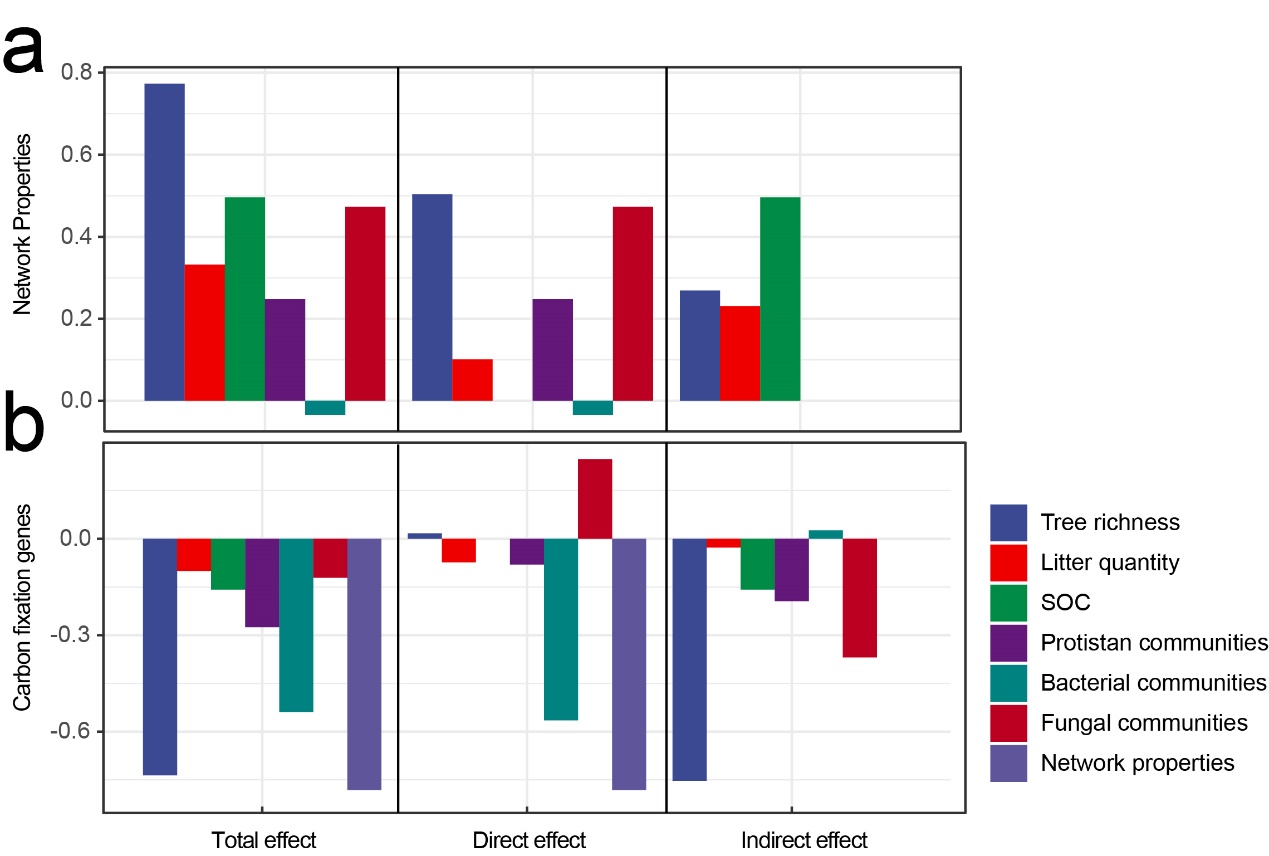
Figure S10.** Direction of total, direct and indirect effects of tree richness, fungal communities, litters, protistan communities, bacterial communities and SOC on network stability (a) and microbial carbon fixation genes (b).


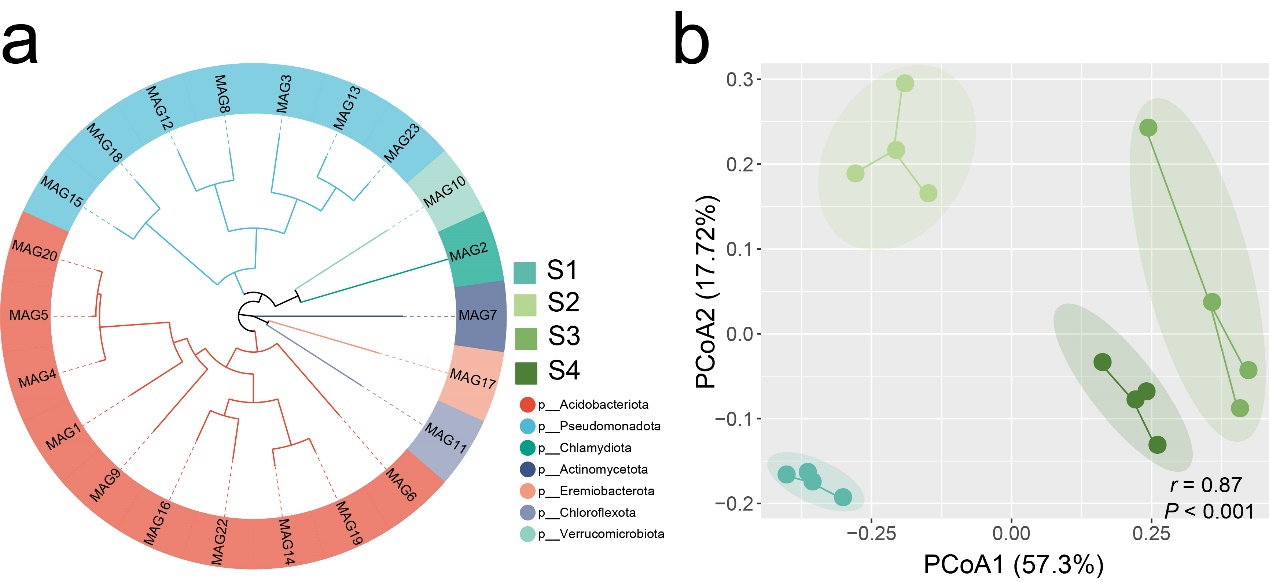


**Figure S11.** Cluster diagram of metagenome-assembled genomes (MAGs) at bacterial phylum level (a), PCoA analysis of MAGs at different stages of forest succession (b). Both analyses are formed from 22 bacterial MAGs. Pine Forest (S1), Broadleaf Forest (S2), elder Mixed Pine and Broadleaf Forest (S3), and Monsoon Evergreen Broadleaf Forest (S4).


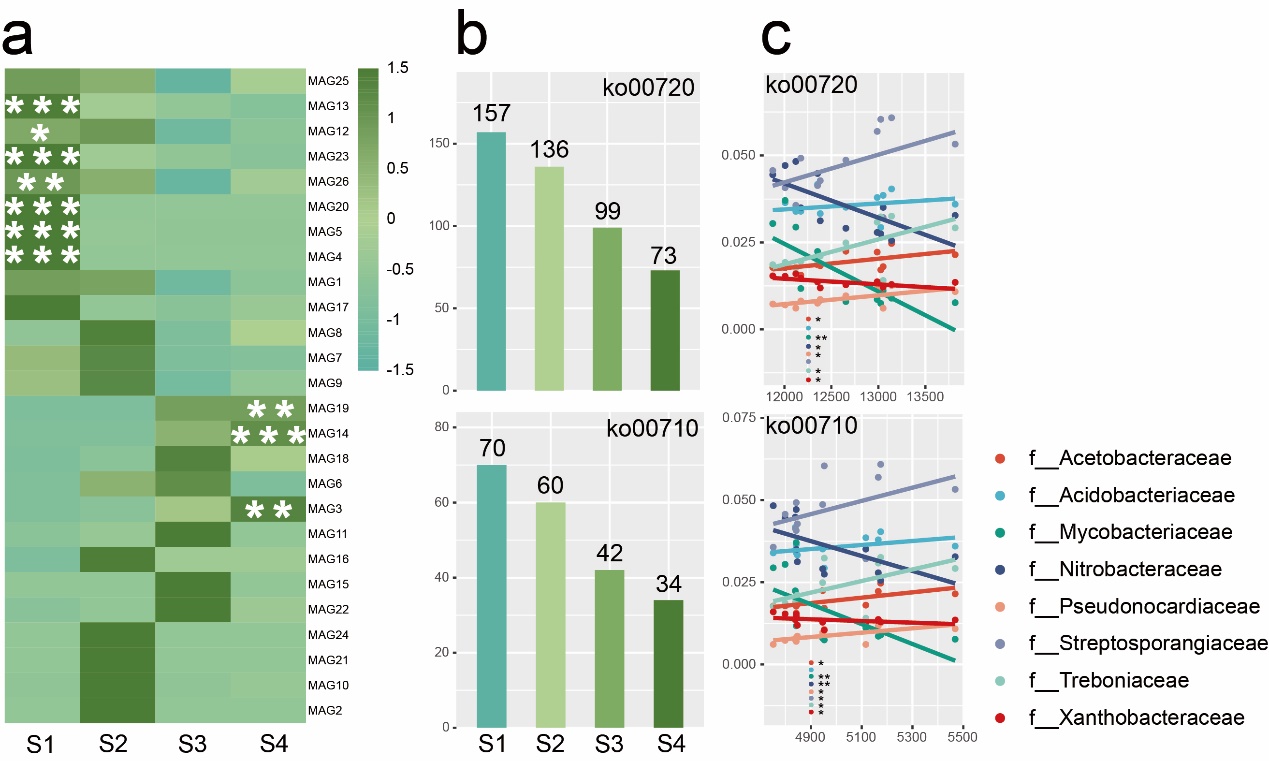


**Figure S12.** Heatmaps of MAG abundance (a); number of related genes detected in the ko00720 and ko00710 pathways (b); linear regression analysis of relationship between family level MAGs and functional genes associated with the ko00720 and ko00710 pathways (c).

**References**

1. Liu, X. *et al.* Plant diversity and species turnover co-regulate soil nitrogen and phosphorus availability in Dinghushan forests, southern China. *Plant Soil* **464**, 257–272 (2021).

2. Bartram, A. K., Lynch, M. D. J., Stearns, J. C., Moreno-Hagelsieb, G. & Neufeld, J. D. Generation of Multimillion-Sequence 16S rRNA Gene Libraries from Complex Microbial Communities by Assembling Paired-End Illumina Reads. *Appl. Environ. Microbiol.* (2011) doi:10.1128/AEM.02772-10.

3. Blaalid, R. *et al.* ITS1 versus ITS2 as DNA metabarcodes for fungi. *Mol. Ecol. Resour.* **13**, 218–224 (2013).

4. Sun, H. *et al.* Novel insights into the rhizosphere and seawater microbiome of Zostera marina in diverse mariculture zones. *Microbiome* **12**, 27 (2024).

5. Bolyen, E. *et al.* Reproducible, interactive, scalable and extensible microbiome data science using QIIME 2. *Nat Biotechnol* **37**, 852–857 (2019).

6. Callahan, B. J. *et al.* DADA2: High-resolution sample inference from Illumina amplicon data. *Nat Methods* **13**, 581–583 (2016).

7. Weinstein, M. M., Prem, A., Jin, M., Tang, S. & Bhasin, J. M. FIGARO: An efficient and objective tool for optimizing microbiome rRNA gene trimming parameters. 610394 Preprint at https://doi.org/10.1101/610394 (2019).

8. Edgar, R. C. UPARSE: highly accurate OTU sequences from microbial amplicon reads. *Nat. Methods* **10**, 996-+ (2013).

9. Pruesse, E. *et al.* SILVA: a comprehensive online resource for quality checked and aligned ribosomal RNA sequence data compatible with ARB. *Nucleic Acids Res* **35**, 7188–7196 (2007).

10. Nilsson, R. H. *et al.* The UNITE database for molecular identification of fungi: handling dark taxa and parallel taxonomic classifications. *Nucleic Acids Res* **47**, D259–D264 (2019).

11. Guillou, L. *et al.* The Protist Ribosomal Reference database (PR2): a catalog of unicellular eukaryote Small Sub-Unit rRNA sequences with curated taxonomy. *Nucleic Acids Res* **41**, D597–D604 (2013).

12. Shenhav, L. *et al.* FEAST: fast expectation-maximization for microbial source tracking. *Nat Methods* **16**, 627–632 (2019).

13. Ning, D., Deng, Y., Tiedje, J. M. & Zhou, J. A general framework for quantitatively assessing ecological stochasticity. *Proc. Natl. Acad. Sci. USA.* **116**, 16892–16898 (2019).

14. Liaw, A. & Wiener, M. Classifcation and Regression by randomForest. *R Journal* **2/3**, (2002).
